# Long-Term Outcomes of Adjuvant Trastuzumab for 9 Weeks or 1 Year for *ERBB2*-Positive Breast Cancer: A Secondary Analysis of the SOLD Randomized Clinical Trial

**DOI:** 10.1001/jamanetworkopen.2024.29772

**Published:** 2024-08-26

**Authors:** Heikki Joensuu, Judith Fraser, Hans Wildiers, Riikka Huovinen, Päivi Auvinen, Meri Utriainen, Kenneth K. Villman, Päivi Halonen, Helena Granstam-Björneklett, Minna Tanner, Liisa Sailas, Taina Turpeenniemi-Hujanen, Jeffrey Yachnin, Teppo Huttunen, Patrick Neven, Peter Canney, Vernon J. Harvey, Pirkko-Liisa Kellokumpu-Lehtinen, Henrik Lindman

**Affiliations:** 1 Department of Oncology, Helsinki University Hospital and University of Helsinki, Helsinki, Finland; 2 Beatson West of Scotland Cancer Centre, Glasgow, United Kingdom; 3 Multidisciplinary Breast Centre, University Hospitals Leuven, Leuven, Belgium; 4 Turku University Hospital, Turku, Finland; 5 Department of Oncology, Kuopio University Hospital, Kuopio, Finland; 6 Örebro University Hospital, Örebro, Sweden; 7 Västerås Central Hospital, Västerås, Sweden; 8 Department of Oncology, Tampere University Hospital and Tampere University, Tampere, Finland; 9 Vaasa Central Hospital, Vaasa, Finland; 10 North Karelia Central Hospital, Joensuu, Finland; 11 Department of Oncology and Radiotherapy, Oulu University Hospital, Oulu, Finland; 12 Center for Clinical Cancer Studies, Karolinska University Hospital, Stockholm, Sweden; 13 EstiMates Ltd, Turku, Finland; 14 Auckland City Hospital, Auckland, New Zealand; 15 Department of Immunology, Genetics and Pathology, Uppsala University Hospital, Uppsala, Sweden

## Abstract

**Importance:**

The standard adjuvant treatment for patients with *ERRB2*-positive breast cancer is chemotherapy plus 1 year of trastuzumab. Shorter durations of trastuzumab administration improve cardiac safety, but more information is needed about their effect on survival.

**Objective:**

To compare survival outcomes after 9-week vs 1-year administration of trastuzumab with the same adjuvant chemotherapy.

**Design, Setting, and Participants:**

This post hoc secondary analysis of an open-label, multicenter, noninferiority-design randomized clinical trial included women aged 18 years or older with early *ERBB2*-positive, axillary node–negative or axillary node–positive breast cancer who were enrolled from January 3, 2008, to December 16, 2014, at 65 centers in 7 European countries. The current exploratory analysis was conducted after achieving the maximum attainable follow-up data when the last patient enrolled had completed the last scheduled visit in December 2022.

**Intervention:**

Chemotherapy consisted of 3 cycles of docetaxel administered at 3-week intervals followed by 3 cycles of fluorouracil, epirubicin, and cyclophosphamide at 3-week intervals. Trastuzumab was administered in both groups for 9 weeks concomitantly with docetaxel. In the 9-week group, no further trastuzumab was administered after chemotherapy, whereas in the 1-year group, trastuzumab was continued after chemotherapy to complete 1 year of administration.

**Main Outcomes and Measures:**

The primary objective was disease-free survival (DFS). Distant DFS and OS were secondary objectives. Survival between groups was compared using the Kaplan-Meier method and log-rank test or univariable Cox proportional hazards regression.

**Results:**

Among the 2174 women analyzed, median age was 56 years (IQR, 48-64 years). The median follow-up time was 8.1 years (IQR, 8.0-8.9 years); 357 DFS events and 176 deaths occurred. Trastuzumab for 9 weeks was associated with shorter DFS compared with trastuzumab for 1 year (hazard ratio [HR], 1.36; 90% CI, 1.14-1.62); 10-year DFS was 80.3% in the 1-year group vs 78.6% in the 9-week group. The 5-year and 10-year OS rates were comparable between the 9-week and 1-year groups (95.0% vs 95.9% and 89.1% vs 88.2%, respectively; HR for all time points, 1.20; 90% CI, 0.94-1.54). In multivariable analyses, 9-week treatment was associated with shorter DFS compared with 1-year treatment (HR for recurrence or death, 1.36; 95% CI, 1.10-1.68; *P* = .005), but there was no between-group difference in OS (HR, 1.22; 95% CI, 0.90-1.64; *P* = .20). Only 4 patients (0.2%) died of a cardiac cause.

**Conclusions and Relevance:**

In this secondary analysis of a randomized clinical trial, 1-year vs 9-week adjuvant trastuzumab was associated with improved DFS among patients with *ERRB2*-positive breast cancer receiving chemotherapy, but there was no significant difference in OS between the groups.

**Trial Registration:**

ClinicalTrials.gov Identifier: NCT00593697

## Key Points

**Question:**

Is brief administration of trastuzumab for 9 weeks with chemotherapy associated with survival similar to that of identical chemotherapy plus 1 year of trastuzumab in the adjuvant treatment of *ERBB2*-positive breast cancer?

**Findings:**

In this secondary analysis of a randomized clinical trial that included 2174 participants, after completion of 8 years of follow-up in the trial, patients assigned to 1 year of trastuzumab had significantly longer disease-free survival compared with those receiving 9 weeks of trastuzumab.

**Meaning:**

The findings suggest that 1 year vs 9 weeks of adjuvant trastuzumab is associated with improved disease-free survival after completed patient follow-up.

## Teaser

This secondary analysis of a randomized clinical trial compares 8-year survival outcomes of 9-week vs 1-year trastuzumab administered concomitantly with chemotherapy among patients with *ERBB2*-positive breast cancer.

## Introduction

The standard adjuvant treatment for patients with localized *ERBB2*-positive breast cancer is chemotherapy plus 1 year of trastuzumab.^1^ Trastuzumab is generally well tolerated, but congestive heart failure may occur, making cardiac function monitoring mandatory.^2,3^

The 1-year duration of adjuvant trastuzumab has been compared with 6-month duration^4,5,6^ or 9-week duration^7,8,9^ in 5 randomized clinical trials that each used noninferiority testing. Noninferiority to 1-year trastuzumab was demonstrated in only 1 of the trials,^6^ but all trials reported better cardiac safety with the shorter regimen.

The multicenter Synergism or Long Duration (SOLD) trial compared 9-week adjuvant trastuzumab plus chemotherapy (9-week group) with an otherwise similar regimen except that trastuzumab was administered after completion of chemotherapy for up to 1 year (1-year group).^7^ In the SOLD trial, trastuzumab was administered for 9 weeks concomitantly with docetaxel in both groups since docetaxel may potentiate trastuzumab efficacy.^10^ In the primary analysis of the SOLD trial, reported after a median patient follow-up time of 5.2 years, when 245 disease-free survival (DFS) events and 102 overall survival (OS) events had accumulated, the 9-week regimen was not noninferior to the 1-year regimen.^7^

We report herein the results of a secondary analysis of the SOLD trial after longer follow-up with more survival events. The current explorative analysis was conducted after achieving the maximum patient follow-up attainable in the trial.

## Methods

### Patients

This post hoc secondary analysis of the SOLD randomized clinical trial (NCT00593697) included patients (aged ≥18 years, with World Health Organization performance score of 0 or 1) with histologically confirmed localized *ERBB2*-positive breast cancer; patients with a history of distant metastases or neoadjuvant therapy were excluded.^7^ The trial protocol (Supplement 1) was approved by the relevant independent ethics committees and medical authorities in the countries where the 65 participating study centers were located (Belgium, Finland, Iceland, New Zealand, Serbia, Sweden, and the United Kingdom). The patients signed informed consent before study inclusion. This report followed the Consolidated Standards of Reporting Trials (CONSORT) reporting guideline for randomized clinical trials.

### Study Procedures

In the SOLD trial, after staging, central randomization (1:1) was done using estrogen receptor (ER) status, axillary nodal status, the *ERBB2* assay method, and the study site as stratification factors. Chemotherapy consisted of 3 cycles of intravenous docetaxel (80 mg/m^2^ or 100 mg/m^2^) given concomitantly with intravenous or subcutaneous trastuzumab, followed by 3 cycles of intravenous fluorouracil (600 mg/m^2^), epirubicin (75 mg/m^2^), and cyclophosphamide (600 mg/m^2^); all 6 cycles were administered at 3-week intervals.^7^ Concomitant trastuzumab was administered intravenously either weekly (first dose, 4 mg/kg; subsequently, 2 mg/kg) or at 3-week intervals (first dose, 8 mg/kg; subsequently, 6 mg/kg) or subcutaneously at 3-week intervals (600 mg, regardless of the body weight). In the 1-year group, intravenous or subcutaneous trastuzumab was administered at 3-week intervals 14 times after stopping chemotherapy. In the 9-week group, no further trastuzumab was administered after chemotherapy.

Endocrine therapy and radiotherapy were given according to the center practice, but adjuvant endocrine therapy was administered for a minimum of 5 years after completion of chemotherapy when cancer was considered ER-positive and/or progesterone receptor–positive. Patients were scheduled for follow-up visits for a minimum of 8 years. Imaging examinations were conducted as clinically indicated during the follow-up period.

### Statistical Analyses

The primary analysis of the SOLD trial was carried out after a median patient follow-up time of 5.2 years based on a preplanned landmark analysis when the last patient enrolled had been followed up for 2 years after the date of randomization on December 31, 2016, and when 245 DFS events and 102 OS events had accumulated.^7^ In the current post hoc secondary analysis of the trial, December 31, 2022, was set as the data collection cutoff date since the last protocol-mandated follow-up visit of the last patient entered in the SOLD trial occurred in December 2022.

The primary objective, DFS, was defined as the time between the date of randomization and date of detection of invasive cancer or death. Secondary objectives included distant DFS (the period from randomization to the date of first detection of distant recurrence of breast cancer or death) and OS (the time from randomization to the date of death).

The SOLD trial^7^ was originally designed as a superiority trial but was amended to a noninferiority trial since it seemed unlikely that DFS in the 9-week arm could be superior to that in the 1-year arm, as severe trastuzumab-related cardiac events were only infrequently observed in other trials^11,12^ that had evaluated adjuvant trastuzumab. Assuming an estimated accrual time of 7.5 years, a relative noninferiority margin of 1.3, 1-sided testing, and a dropout rate of 3%, the estimated final sample size was 2168 patients (Supplement 1). The margin of 1.3 was based on assuming 85% 5-year DFS in the 1-year group, corresponding to an absolute DFS difference of 4% between the groups.

Survival analyses in the current study were based on the intention-to-treat population. Survival between the groups was compared using the Kaplan-Meier life table method and the log-rank test or a univariable Cox proportional hazards regression model; hazard ratios (HRs) were calculated using a univariable Cox proportional hazards regression model. Interactions between subgroup variables and the treatment group were analyzed using the Cox proportional hazards regression model. Survival analysis results between the groups are provided using a 90% 2-sided CI since this corresponds to the upper limit of the 1-sided 95% CI used in the evaluation of noninferiority and is consistent with the earlier reporting.^7^ The *P* values are 2-sided, unadjusted for multiple testing, and should be interpreted as exploratory; *P* <.05 was considered significant. Statistical analyses were performed with SAS, version 9.4 (SAS Institute, Inc).

## Results

### Patients and Follow-Up

A total of 2176 patients were enrolled between January 3, 2008, and December 16, 2014 (eFigure 1 in Supplement 2). Two patients were excluded from further analysis due to presence of distant metastases on the date of randomization. The median age of the 2174 patients analyzed (1085 [49.9%] in the 9-week group and 1089 [50.1%] in the 1-year group) was 56 years (IQR, 48-64 years). Most patients had node-negative cancer (647 [59.6%] in the 9-week group and 649 [59.6%] in the 1-year group). Patient and tumor characteristics are summarized in the Table.

**Table.** Patient and Breast Cancer Characteristics

Characteristic	Patients^a^	
	9-wk Group (n = 1085)	1-y Group (n = 1089)
Age, median (IQR), y	56 (49-64)	56 (48-63)
WHO performance status		
0	975 (89.9)	963 (88.4)
1	102 (9.4)	112 (10.3)
Not available	8 (0.7)	14 (1.3)
Breast tumor size, mm		
≤10	129 (11.9)	155 (14.2)
11-20	473 (43.6)	453 (41.6)
21-50	447 (41.2)	452 (41.5)
>50	36 (3.3)	29 (2.7)
Positive axillary nodes, No.		
0	647 (59.6)	649 (59.6)
1-3	322 (29.7)	320 (29.4)
>3	116 (10.7)	120 (11.0)
Stage		
I	429 (39.5)	432 (39.7)
II	527 (48.6)	526 (48.3)
III	129 (11.9)	131 (12.0)
Histologic grade		
1	26 (2.4)	27 (2.5)
2	340 (31.3)	327 (30.0)
3	714 (65.8)	731 (67.1)
Not available	5 (0.5)	4 (0.4)
Histologic type		
Ductal	1000 (92.2)	1000 (91.8)
Other	83 (7.6)	88 (8.1)
Not available	2 (0.2)	1 (0.1)
ER status		
Positive	711 (65.5)	723 (66.4)
Negative	374 (34.5)	366 (33.6)
*ERBB2* status		
Positive	1078 (99.4)	1084 (99.5)
Negative	0	1 (0.1)
Unconfirmed	7 (0.6)	4 (0.4)

Abbreviations: ER, estrogen receptor; WHO, World Health Organization.

^a^ Data are presented as number (percentage) of patients unless otherwise indicated.

The median follow-up time of the study participants was 8.1 years (IQR, 8.0-8.9 years) after randomization; 3 were lost to follow-up. Of the 357 DFS events recorded, 202 (56.6%) and 155 (43.4%) occurred in the 9-week and 1-year groups, respectively, and of the 176 deaths, 95 (54.0%) and 81 (46.0%), respectively.

### DFS

The patients assigned to the 9-week group had shorter DFS than those assigned to the 1-year group (HR for recurrence or death, 1.36; 90% CI, 1.14-1.62; exploratory 2-sided log-rank superiority testing *P* = .004) (Figure, A). Five-year DFS was 90.7% in the 1-year group and 87.7% in the 9-week group, and 10-year DFS was 80.3% and 78.6%, respectively.

**Figure.**
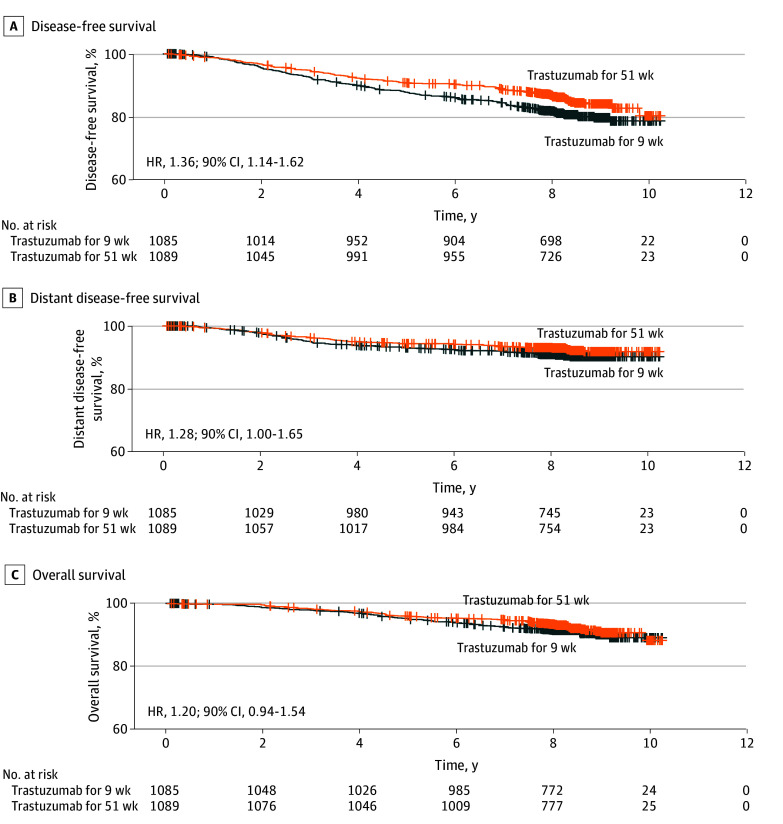
Survival Outcomes After the Date of Randomization Hash marks indicate patients without an event. HR indicates hazard ratio.

When DFS was analyzed in subgroups defined by the stratification factors used at randomization or factors predefined in the statistical analysis plan (Supplement 1), a significant interaction was detected between the docetaxel starting dose (80 mg/m^2^ or 100 mg/m^2^) and the differences in DFS by treatment group (*P* = .007 for interaction) (eFigure 2 in Supplement 2). In a multivariable Cox proportional hazards regression model, a high number (≥4) of positive axillary nodes (HR, 2.28; 95% CI, 1.65-3.15; *P* < .001), disease stage of II or III vs I (HR, 1.53; 95% CI, 1.13-2.08; *P* = .006), and being in the 9-week group (HR, 1.36; 95% CI, 1.10-1.68; *P* = .005) were associated with shorter DFS, whereas ER status, age, and the docetaxel starting dose were not (eTable 1 in Supplement 2).

### Distant DFS and OS

Distant DFS and OS did not differ significantly between the groups (distant DFS: HR, 1.28 [90% CI, 1.00-1.65]; exploratory 2-sided log-rank superiority testing *P* = .10; OS: HR, 1.20 [90% CI, 0.94-1.54]; exploratory 2-sided log-rank superiority testing *P* = .22) (Figure, B and C). In the 9-week group, the 5-year and 10-year OS rates were 95.0% and 89.1%, respectively, and in the 1-year group, these were 95.9% and 88.2%, respectively. In subgroup analyses for OS, no interactions were detected between the factors tested (ER status, docetaxel dose, positive axillary lymph nodes, age, and disease stage) and the treatment group (eFigure 3 in Supplement 2). In a multivariable analysis, the number of positive axillary nodes (HR, 2.77; 95% CI, 1.79-4.30; *P* < .001), age at study entry (HR, 1.03; 95% CI, 1.01-1.05; *P* < .001), and disease stage (HR, 1.93; 95% CI, 1.22-3.03; *P* = .005) were independently associated with the risk of death, whereas cancer ER status, the docetaxel starting dose, and the treatment group (HR, 1.22; 95% CI, 0.90-1.64; *P* = .20) were not (eTable 2 in Supplement 2). Survival outcomes for axillary nodal metastasis categories are provided in eFigure 4 in Supplement 2.

### Cardiac Deaths

Four patients (0.2%) died of a cardiac cause (coronary artery thrombosis and/or myocardial infarction, 3 [75.0%]; heart failure, 1 [25.0%]). Three of these patients (75.0%) had received trastuzumab for 9 weeks.

## Discussion

In this secondary analysis of the SOLD trial, during the follow-up period, 357 DFS events and 176 deaths occurred, which are substantially higher numbers compared with the those in the first analysis of the SOLD trial.^7^ There was longer DFS in the 1-year group, but distant DFS and OS did not differ significantly between the groups. These results are comparable to those observed in the first analysis of the trial,^7^ supporting robustness and stability of the findings. In multivariable analyses, treatment group was independently associated with DFS but not with OS.

These observations mostly agree with those reported from the ShortHER trial,^8,9^ which is, to our knowledge, the only other randomized clinical trial that compared the 9-week and 1-year durations in a comparable patient population. In the ShortHER trial, noninferiority of the 9-week regimen could not be claimed, but there was numerically little difference in DFS or OS between the groups. Unlike the ShortHER trial, we did not find an interaction between the treatment group and the axillary nodal metastasis category.

Few patients died of a cardiac cause. This observation is consistent with findings in other trials in which death from cardiac failure was uncommon in patients treated with trastuzumab.^6,13,14^

Potential advantages of the 9-week regimen include little need for cardiac monitoring,^7,15^ fewer visits required for treatment administration, and lower cost. Access to trastuzumab is still a major barrier to care, particularly in low-income and lower-middle-income countries.^16^

### Strengths and Limitations

The SOLD trial design may be considered a strength of this study since chemotherapy was identical in the 2 arms and randomization took place before starting the systemic treatments. Study limitations include the follow-up time, which may have been too short for capturing all recurrences and breast cancer–related deaths despite *ERBB2*-positive breast cancers tending to recur earlier than ER-positive, *ERBB2*-negative breast cancers and usually within the first 8 years since their detection.^17^ Most patients (59.6%) had node-negative cancer; this proportion is slightly larger but in the same order of magnitude as in other large randomized clinical trials that have evaluated shorter than 1-year duration of adjuvant trastuzumab, in which 54% to 59% of the patients had node-negative disease.^5,6,8,9^

## Conclusions

In this secondary analysis of the SOLD trial with longer follow-up time and more events in the analysis, patients allocated to 1 year of adjuvant trastuzumab had longer DFS than patients receiving 9 weeks of adjuvant trastuzumab. Yet, there were no significant differences in the 5-year and 10-year distant DFS and OS rates between the groups. The 9-week regimen may be an option for patients who may not tolerate 1-year trastuzumab or who cannot afford it.

## Notes

Supplement 1.
Trial Protocol

Supplement 2. **eTable 1.** Multivariable Cox Proportional Hazards Regression Model for Disease-Free Survival **eTable 2.** Multivariable Cox Proportional Hazards Regression Model for Overall Survival **eFigure 1.** CONSORT Diagram of the Trial **eFigure 2.** Disease-Free Survival in Prespecified Subgroups **eFigure 3.** Overall Survival in Prespecified Subgroups **eFigure 4.** Disease-Free Survival and Overall Survival by Treatment Group in Axillary Nodal Metastasis Categories

Supplement 3.
Data Sharing Statement
