# Supplementary material for: Long-Term Outcomes of Adjuvant Trastuzumab for 9 Weeks or 1 Year for ERBB2-Positive Breast Cancer: A Secondary Analysis of the SOLD Randomized Clinical Trial
Source: JAMA Netw Open. 2024 Aug 26;7(8):e2429772. doi: 10.1001/jamanetworkopen.2024.29772 (PMC12507463; doi:10.1001/jamanetworkopen.2024.29772)
Supplement: Supplement 1. — Trial Protocol [file jamanetwopen-e2429772-s001.pdf]

CLINICAL STUDY PROTOCOL

A randomized phase III study comparing trastuzumab plus docetaxel (HT) followed by 5-FU, epirubicin, and cyclophosphamide (FEC) to the same regimen followed by single-agent trastuzumab as adjuvant treatments for early breast cancer

The Synergism Or Long Duration (SOLD) Study

Protocol number FBCSG-01-2007

EudraCT number 2007-002016-26

Study register identification number: NCT00593697

Draft Version 5.4 (final); Amended v. 5.41 (Feb 21, 2014)

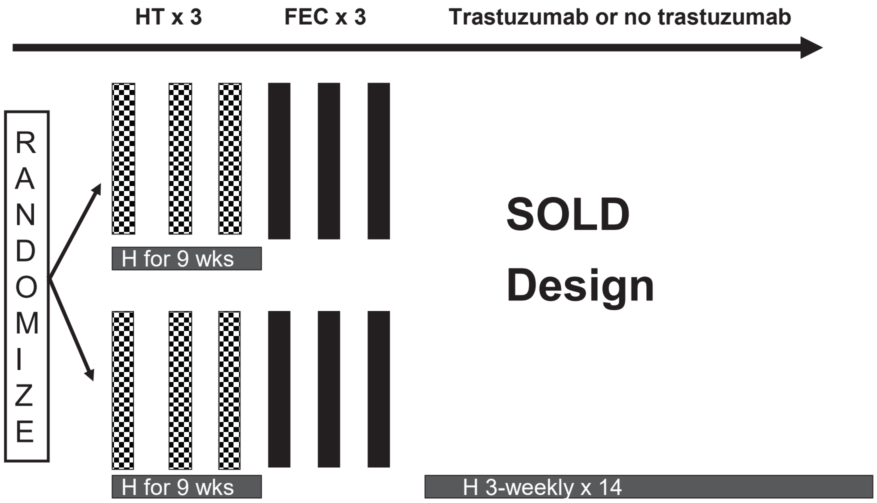

Coordinating investigator
Heikki Joensuu, M.D., Ph.D., Department of Oncology, Helsinki University Central Hospital, Haartmaninkatu 4 (P.O. Box 180), FIN-00029 Helsinki, FINLAND.
Tel. +358-9-471 73208; Email heikki.joensuu@hus.fi

PROTOCOL OUTLINE

TITLE	**A randomized phase III study comparing trastuzumab plus docetaxel (HT) followed by 5-FU, epirubicin and cyclophosphamide (FEC) to the same regimen followed by single-agent trastuzumab as adjuvant treatments for early breast cancer**<br><br>**The "Synergism Or Long Duration" (SOLD) Study**
SPONSOR	The Finnish Breast Cancer Group
INDICATION	Female patients with invasive HER2-positive early breast cancer with no distant metastases, who have a high risk of distant recurrence within 5 years from the time of diagnosis.
OBJECTIVES	**Primary:** To compare disease-free survival (DFS) between the 2 treatment arms.<br>**Secondary:**<br>To compare<br>• Overall survival<br>• Distant disease-free survival<br>• Cardiac event-free DFS<br>• Left ventricle ejection fractions<br>• Adverse event rate using the Common Terminology Criteria for Adverse Events (CTCAE) version 3.0<br>• Quality of life
TRIAL DESIGN	An open-label, 2-arm, prospective, randomized, multi-center phase III trial.<br>Patients diagnosed with early breast cancer with a high risk of disease recurrence will be randomly allocated to one of the following 2 arms in a 1:1 ratio:

A. Weekly or 3-weekly trastuzumab plus 3-weekly docetaxel (3 cycles) (HT) → 3-weekly $FE_{75}C$ (3 cycles) (HT x3→$FE_{75}C$ x3)

B. Weekly or 3-weekly trastuzumab plus 3-weekly docetaxel (3 cycles) (HT) → 3-weekly $FE_{75}C$ (3 cycles) → trastuzumab to complete 1 year (14 3-weekly infusions) (HT x3→$FE_{75}C$ x3→ H_{3wkly} x14)

Participants will be followed up for a minimum of 8 ~~5~~ years post-randomization or until death.

Locoregional radiotherapy will be given according to the institutional practice.

Patients with ER and/or PgR positive disease will receive adjuvant endocrine therapy for a minimum of 5 years.

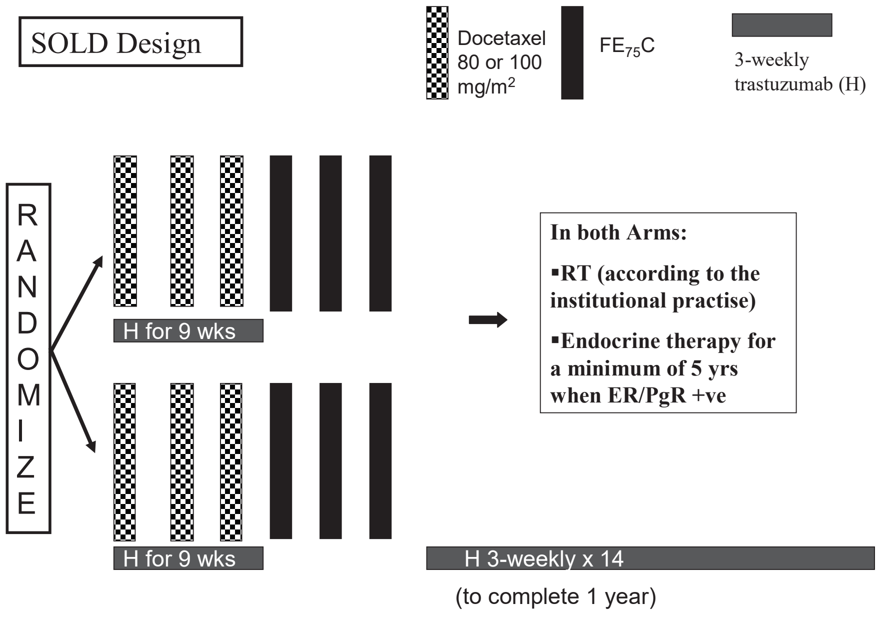

NUMBER OF PATIENTS	2,168 ~~3,000~~ patients (1084 ~~1,500~~ patients per arm) to be randomized.
RANDOMIZATION	Randomization, stratified by the axillary nodal status (pN0 or pN1 vs. pN2 or pN3), the method of HER2 assessment (*in situ* hybridization or

	immunohistochemistry), hormone receptor expression (ER positive or ER negative) and the study center will be carried out using a central computer-generated randomization system.
TARGET POPULATION	**Inclusion Criteria**<br>To be eligible for inclusion in the study, each patient must fulfill each of the criteria below.<br>1. Patient has provided a written informed consent prior to study-specific screening procedures, with the understanding that she has the right to withdraw from the study at any time, without prejudice.<br>2. Woman $\geq$ 18 years of age.<br>3. Histologically confirmed invasive breast cancer.<br>4. HER2-positive breast cancer (preferably assessed with CISH or FISH; if not available with immunohistochemistry 3+)<br>5. A high risk of breast cancer recurrence with *one* of the following:<br>i) Pathological N0 with the longest invasive tumor diameter >5 mm<br>ii) Histologically confirmed regional node positive disease (pN+; nodal isolated tumor cells/cell clusters < 0.2 mm in diameter (ITP) are not counted as a metastasis)<br><br>**Exclusion Criteria**<br>Patients who fulfill any of the following criteria will be excluded:<br>1. Presence of distant metastases.<br>2. Inflammatory breast cancer.<br>3. pT1bN0M0 (i.e. the longest tumor diameter 6 to 10 mm, node-negative) *and* histological grade 1.<br>4. Clinically significant (i.e. active) cardiac disease (e.g. congestive heart failure, symptomatic coronary artery disease and cardiac arrhythmia not well controlled with medication) or myocardial infarction within the last 12 months.<br>5. Left ventricular ejection fraction less than 50% (or under the institutional normal reference range)

assessed by echocardiography or isotope cardiography.

6. ER, PgR and HER-2 status (via *in situ* hybridization or immunohistochemistry) not determined.
7. Primary systemic cancer therapy (neoadjuvant chemotherapy or endocrine therapy) has been administered prior to breast surgery.
8. The WHO performance status > 1.
9. Pregnant or lactating women.
10. Women of childbearing potential unless using a reliable and appropriate contraceptive method. Women must have been amenorrheic for at least 12 months prior to study entry to be considered postmenopausal and to have no childbearing potential. Women of childbearing potential (menstruating within 12 months of study entry), or with no hysterectomy and age < 55, must have a negative pregnancy test at baseline.
11. More than 12 weeks between breast surgery and date of randomization.
12. Organ allografts with immunosuppressive therapy required.
13. Major surgery (except breast surgery) within 4 weeks prior to study treatment start, or lack of complete recovery from the effects of major surgery.
14. Participation in any investigational drug study within 4 weeks preceding treatment start.
15. Patients with a history of uncontrolled seizures, central nervous system disorders or psychiatric disability judged by the investigator to be clinically significant precluding study participation.
16. History of another malignancy within the last five years except cured basal cell carcinoma of skin or carcinoma *in situ* of the uterine cervix.
17. One or more of the following:
    - Blood hemoglobin $\leq$ 10.0 g/dL, neutrophils < 1.5 x 10^9/L, platelet count < 120 x 10^9/L
    - Serum/plasma creatinine > 1.5 x Upper Limit of Normal (ULN)

- Serum/plasma bilirubin > ULN
- Serum/plasma ALT and/or AST > 1.5 x ULN
- Serum/plasma alkaline phosphatase > 2.5 x ULN

18. Serious uncontrolled infection or other serious uncontrolled concomitant disease.
19. Unwilling or unable to comply with the protocol for the duration of the study.
20. History of hypersensitivity to the investigational products or to drugs with similar chemical structures.
21. Pre-existing motor or sensory neurotoxicity of a severity ≥ grade 2 by CTCAE version 3, unless related to mechanical etiology.

STUDY DRUG REGIMENS

Arm A: HT x 3 → FEC x 3

1) HT:

3 cycles of HT will be administered. Trastuzumab (H) and docetaxel (T) are given on day 1 of a 21-day docetaxel cycle.

Trastuzumab may be given either weekly or 3-weekly in combination with docetaxel. Each center is recommended to ~~must~~ use the same trastuzumab administration schedule (either weekly or 3-weekly) throughout the study.

Weekly trastuzumab: First dose 4 mg/kg, subsequent doses 2 mg/kg; administered intravenously on Cycle Day 1, infused prior to docetaxel.

3-weekly trastuzumab: First dose 8 mg/kg, subsequent 2 doses 6 mg/kg; administered intravenously on Cycle Day 1, infused prior to docetaxel. Alternatively, trastuzumab may be administered subcutaneously 3-weekly at a fixed dose of 600 mg.

Docetaxel: 80 mg/m^2 or 100 mg/m^2 administered as a 60-minute intravenous (IV) infusion (with appropriate pre-medication). Each center must define the starting dose of docetaxel (see protocol p. 30 for options).

2) FEC: 3 cycles will be administered. All 3 agents are given on day 1 of a 21-day cycle.

5-Fluorouracil: 600 mg/m^2 IV

Epirubicin: 75 mg/m^2 IV ($\geq$60 minute infusion)

Cyclophosphamide: 600 mg/m^2 IV

Arm B: HT x 3 $\rightarrow$ FEC x 3 $\rightarrow$H (3 wkly) x 14

3 cycles of HT followed by 3 cycles of FEC are administered as in Arm A.

Single-agent trastuzumab (H) is administered 14 times 3-weekly as follows:

The first dose is 8 mg/kg, the subsequent 13 doses 6 mg/kg. Alternatively, single-agent trastuzumab may be administered subcutaneously at a fixed dose of 600 mg 3-weekly 14 times.

Radiotherapy. Locoregional radiotherapy will be given according to the institutional practice in both arms either prior to or after chemotherapy. When radiotherapy is given after chemotherapy it is recommended to be started within 2 months after the last chemotherapy infusion (the third FEC).

Endocrine therapy. Upon completion of chemotherapy, all patients who have ER positive and/or PgR positive disease will also receive endocrine therapy (aromatase inhibitor/tamoxifen/LHRH agonists) according to the institutional practice for a minimum of 5 years. Endocrine therapy should be initiated within 3 months of completing chemotherapy.

ASSESSMENTS OF:

EFFICACY

Primary:

- Disease-free survival (DFS) (endpoints include locoregional recurrence of cancer, distant recurrence of cancer, contralateral BC, second cancer, death from an unrelated cause).

Secondary:

- Overall survival
- Distant disease-free survival

	• Cardiac event-free DFS (endpoints include congestive heart failure requiring medication/intervention, myocardial infarction, or cardiac or coronary artery surgery/stenting after study entry)<br>• LVEF (left ventricle ejection fraction)<br>• Adverse effects and safety<br>• Quality of life between the allocation groups
SAFETY	Adverse events and laboratory tests, graded according to the CTCAE (version 3.0), premature withdrawals.<br>Cardiac safety monitoring (LVEF either with echocardiography or isotope cardiography) measured pretreatment, and on Study Weeks 18, 31, 43, and 61, and Study Month 36 after randomization. Each patient needs to be monitored with the same method throughout the study.<br>An Independent Safety Monitoring Committee will be nominated.
STATISTICAL CONSIDERATIONS	This is a noninferiority ~~superiority~~ study regarding the main endpoint (DFS). Approximately 2,168 ~~3,000~~ patients need to be entered to the study (power 0.8, 1- ~~2-~~sided alpha 0.05).

SCHEDULE OF ASSESSMENTS AND PROCEDURES

**Tests/ Observations**	**Screening**		**Chemotherapy phase**	**Post-chemotherapy phase**	**End of Treatment**	**Post-Therapy Follow-up**
	**Completed no more than (time) prior inclusion**		**Chemotherapy cycles HTx3 followed by FECx3 (Arm A and Arm B)**	**Follow-up (Arm A) or Single-Agent Trastuzumab (Arm B)**	**(Arm A and Arm B)**	**(Arm A and Arm B)**
**Study Week/Month**			**Weeks 1, 4, 7, 10, 13, and 16**	**Weeks (±1 week) 19, 22,25,28,31,34,37,40, 43,46,49,52,55,58**	**Week 61 (±2 weeks)**	**Month (±1 month) 24, 36, 48, 60, 72, 84, 96 and 108 ~~84~~**
Informed consent (a)	x	28 days				
Inclusion/ exclusion criteria	x	28 days				
Medical history&Demographics (b)	x	28 days				
Cancer/ treatment history (c)	x	28 days				
Physical examination incl. vital signs (d)	x	28 days	x	Weeks (±2 wks) 31 and 43	x	x
Existing signs & symptoms	x	28 days	x	Weeks (±2 wks) 31 and 43	x	x
ER, PgR; HER2 analysis (FISH or CISH; if not available, IHC)	x					
Hematology (e); Biochemistry (f)	x	7 days	Day -3 to 0 before each cycle	Weeks (±2 wks) 31 and 43	x	x[g]
Pregnancy test (h)	x	7 days	As clinically indicated			
LVEF monitoring (i)	x	28 days	Week 18 ± 2 weeks	Weeks (±2 wks) 31 and 43	x	Month 36±1 mo
Bone scan/x-ray; chest CT/x-ray; CT/ MRI/ US of liver if ≥pN4+, (j); electrocardiogram (ECG)	x	28 days	As clinically indicated			
Adverse events			x	x	x	
SAE reporting			x			
Research blood/ serum sample (optional) (k)	x	7 days			x	x
Quality of life	x	28 days	Cycles 3 and 6, Week 18± 2 wks	Weeks (±2 wks) 31 and 43	x	Month 36±1 mo

<table>
<tr><td>Tumor tissue collection for research/HER2 reassessment</td><td></td><td></td><td colspan="3">x</td></tr>
<tr><td>Radiation therapy (l)</td><td></td><td></td><td></td><td>x</td><td></td></tr>
<tr><td>Endocrine therapy (m)</td><td></td><td></td><td></td><td colspan="2">x</td></tr>
<tr><td>Follow-up/additional therapies</td><td></td><td></td><td colspan="3">x</td></tr>
</table>

Footnotes:

(a) Written informed consent must be signed and dated by the patient prior any study-specific procedures.
(b) Includes age, menopausal status, race, concomitant diseases and regular medication, history of malignancies.
(c) Includes date of first histological diagnosis, type of surgery, sentinel node biopsy, histological type, histological grade, hormone receptor status and assay type (ER, PgR), HER2 assay type, pTNM classification, number of metastatic nodes.
(d) Includes physical examination, height (measured only once), weight, WHO performance status, blood pressure, and pulse rate.
(e) Includes blood haemoglobin, and leukocyte, neutrophil, and platelet counts.
(f) Includes serum or plasma creatinine, bilirubin, ALT and/or AST, and alkaline phosphatase.
(g) Blood cell counts and blood biochemistry measured according to the institutional practice during follow-up (Months 24 to 84)
(h) For women of childbearing potential only.
(i) One evaluation method should be used throughout the study (either echocardiography or isotope cardiography).
(j) Staging examinations are carried out according to the institutional practice. Bone scan or bone x-ray; chest CT or x-ray; CT/ MRI or US of liver are mandatory when ≥pN4+ (4 or more lymph nodes contain cancer), pN3, or when the primary tumor is classified as pT3 or pT4.
(k) 2 mL of serum and 2 mL of plasma (optional; samples are stored at -20˚C or colder).
(l) Administered according to the institutional practise (guidelines provided). Radiation therapy may be given either before or after chemotherapy.
(m) Endocrine therapy for a minimum of 5 years when hormone receptor status is positive (ER and/or PgR positive) according to the institutional practice.

GLOSSARY OF ABBREVIATIONS

5-FU	5-fluorouracil
CISH	chromogenic *in situ* hybridization
FISH	fluorescence *in situ* hybridization
HT	trastuzumab (H)-docetaxel (T)
ALT (SGPT)	alanine aminotransferase<br>serum glutamic pyruvic transaminase
AST (SGOT)	aspartate aminotransferase<br>serum glutamic oxaloacetic transaminase
FEC	5-fluorouracil-epirubicin-cyclophosphamide
GCP	good clinical practice
CHF	congestive heart failure
CMF	cyclophosphamide, methotrexate and 5-fluorouracil
CT	computer tomography
CTCAE	Common Terminology Criteria for Adverse Events
CRF	case report form(s)
HER2	human epidermal growth factor receptor-2
ECG	Electrocardiogram
IHC	Immunohistochemistry
ER	estrogen receptor
PgR	progesterone receptor
LVEF	left ventricular ejection fraction
MRI	magnetic resonance imaging
pN	pathological N (nodal) stage
DFS	disease free survival
RECIST	response evaluation criteria in solid tumors
IV	Intravenous
ULN	upper limit of normal
vs	Versus
SAE	Serious Adverse Event
SUSAR	Suspected Unexpected Serious Adverse Reaction

TABLE OF CONTENTS

Page

1. BACKGROUND AND RATIONALE 16
1.1 Disease Background 16
1.2 Rationale of the SOLD Study 17
2. STUDY OBJECTIVES 19
2.1 Primary Objective 19
2.2 Secondary Objectives 19
3. STUDY DESIGN AND DURATION 20
3.1 Overview of Study Design 20
3.2 Treatment Duration 21
4. RANDOMIZATION PROCEDURE 21
5. STUDY POPULATION 22
5.1 Target Population 22
5.2 Inclusion Criteria 22
5.3 Exclusion Criteria 23
6. SCREENING PROCEDURES 24
6.1 Informed Consent, Eligibility Screening Form 24
6.2 Demographic Data 24
6.3 Medical History 24
6.4 Cancer/Treatment History 25
6.5 Physical Examination And Vital Signs 25
6.6 Laboratory Assessments 25
6.7 Pregnancy Test 26
6.8 Left Ventricle Ejection Fraction (LVEF) And Electrocardiogram 26
6.9 Staging Examinations 26
7. STUDY MEDICATION 27
7.1 Dose And Schedule of Study Medications 27
7.2 Trastuzumab-Docetaxel (HT) 29
7.3 Prophylactic G-CSFs Following Trastuzumab-Docetaxel Administration 31
7.4 5-Fluouracil-Epirubicin-Cyclophosphamide (FEC) 32
7.5 Single-Agent Trastuzumab (H) Following HT/FEC (Arm B) 32
7.6 Use of Prophylactic Antibiotics 33
7.7 Use of Prophylactic Antiemetics 33
7.8 Determination of the Dose Based on BSA 33
7.9 Study Medication Administration Records 33
8. CHEMOTHERAPY DOSE MODIFICATIONS FOR TOXICITY 34
8.1 Trastuzumab-Docetaxel (HT) Dose Modifications for Toxicity 34
8.2 FEC Dose Modifications for Toxicity 35
8.3 Single-Agent Trastuzumab Dose Modifications for Toxicity (Arm B) 36
8.4 Special Notes Regarding Dose Modifications for Toxicity 36
9. WARNINGS AND PRECAUTIONS 37

9.1 Trastuzumab 37
9.2 Docetaxel 38
10. MANAGEMENT OF TOXICITY 39
9.1 Neutropenia 39
9.2 Hypersensitivity Reactions 39
9.3 Grade ≥ 2 Nausea/Vomiting 40
9.4 Fluid Retention 40
9.5 Hepatic Impairment 40
11. RADIATION THERAPY 40
12. ENDOCRINE THERAPY 41
13. CONCOMITANT MEDICATION AND TREATMENT 41
    13.1 Concomitant Medication 41
    13.2 Major Surgery 42
    13.3 Cardiac Treatment 42
    13.4 Other Anticancer Therapies 42
14. DISCONTINUATION OF THERAPY 42
15. PREMATURE WITHDRAWAL 42
16. SAFETY INSTRUCTIONS AND GUIDANCE 43
    16.1 Clinical Adverse Events 43
    16.2 Laboratory Test Abnormalities 43
    16.3 Expected Serious Adverse Reactions 43
    16.4 Suspected Unexpected Serious Adverse Reactions (SUSARs) 44
    16.5 Serious Adverse Events (SAEs) 44
    16.6 Treatment And Follow-Up of Adverse Events 45
    16.7 Pregnancy 45
17. ASSESSMENT OF QUALITY OF LIFE 45
18. RESEARCH SERUM/BLOOD SAMPLES 46
19. RESEARCH TISSUE SAMPLES 46
20. CORRELATIVE BIOLOGICAL STUDIES 46
21. HER2 REASSESSMENT AS QUALITY CONTROL 47
22. STATISTICAL CONSIDERATIONS AND ANALYSIS PLAN 47
    22.1 Primary And Secondary Study Variables 47
    22.2 Analysis Populations 47
    22.3 Primary Efficacy Analysis (DFS) 48
    22.4 Cardiac Event-free DFS 49
    22.5 Distant Disease-Free Survival And Overall Survival 49
    22.6 Adverse Events And Premature Withdrawals 49~~50~~
    22.7 LVEF 50
    22.8 Safety Analyses And Interim Analyses 50
    22.9 Sample Size Considerations 51
    22.10 Replacement Policy 51

23. STUDY DURATION AND DATES 51
24. GENERAL STUDY ADMINISTRATION 51
24.1 Study Steering Committee 52
24.2 Independent Data Safety Monitoring Committee 52
25. ETHICAL ASPECTS AND PARTICIPANT PROTECTION 52
25.1 Declaration of Helsinki, GCP, And Local Regulations 52
25.2 Patient Informed Consent 52
25.3 Institutional Review Board/Ethics Committee 52
25.4 Conditions for Modifying the Protocol 53
25.5 Conditions for Terminating the Study 53
26. STUDY DOCUMENTATION AND CRFs 53
26.1 Investigator's Files/Retention of Documents 53
26.2 Source Documents And Background Data 54
26.3 Audits And Inspections 54
26.4 Case Report Forms 54
26.5 Confidentiality of Trial Documents And Patient Records 54
27. MONITORING OF THE STUDY 54
28. PUBLICATION OF DATA 55
29. REFERENCES 55
30. APPENDICES 58
Appendix 1 - WHO Performance Status 58
Appendix 2 - Nomogram for BSA Determination 59
Appendix 3 - Common Terminology Criteria for Adverse Events v3.0 (CTCAE) 60
Appendix 4 – EuroQol EQ-5D Quality of Life Instrument 61
Appendix 5 – The CIOMS Form 67
Appendix 6 – A Guideline for Radiotherapy Administration 69
Appendix 7 – Contact Addresses 73
Appendix 8 – Selection of aromatase inhibitor for the study in Finland 74
Appendix 9 – Amended power and sample size calculations (Feb. 2014) 74

1. BACKGROUND AND RATIONALE

1.1 Disease Background

Breast cancer is the most common type of cancer of women in industrialized countries. Surgery with or without radiotherapy can control local-regional disease in the majority of patients, but approximately a third of breast cancer patients will ultimately die from the disease. Systemic adjuvant cancer therapy, administered either before or following surgery, is based on the rationale that subclinical tumor remaining after surgery can be more easily eradicated than overt macroscopical recurrence of cancer. Adjuvant chemotherapy has been established effective in the treatment of breast cancer based on numerous randomized trials and several meta-analyses including data collected worldwide.[1] Long-term follow-up results from randomized trials indicate that some breast cancer patients can be cured of the disease by adjuvant systemic therapy.[2]

Systemic adjuvant chemotherapy has evolved and improved in efficacy with time. In general, anthracycline-containing chemotherapy regimens are more effective than CMF particularly when cancer cells express HER2/topoisomerase II-alpha,[1,3,4] and taxane-containing regimens may be more effective than comparable non-taxane regimens.[5] At present, patients who have node-positive breast cancer, or cancer with other features suggesting a high risk for cancer recurrence such as amplification of the HER2 (erbB2) gene, are often treated with combination chemotherapy that includes a taxane and an anthracycline. Adjuvant endocrine therapy administered for a minimum of 5 years following completion of chemotherapy is considered standard whenever breast cancer is hormone receptor positive.[6]

Approximately 20% of all breast cancers express HER2, a transmembrane tyrosine kinase with no known ligand. HER2-positive breast cancers are generally associated with less favorable outcome than HER2-negative cancers.[7,8] Even small node-negative HER2-positive cancers have a relatively high risk for distant recurrence.[9-11]

Trastuzumab is a humanized monoclonal antibody that targets HER2. Results based on limited follow-up from large prospective, randomized trials indicate that adjuvant administration of trastuzumab for 12 months reduces substantially the risk of breast cancer recurrence, but at the expense of some cardiotoxicity.[12,13] According to one of these trials, concomitant administration of trastuzumab with a taxane (weekly paclitaxel) was more effective that sequential administration of the same drugs.[12] However, single-agent trastuzumab administered after completion of chemotherapy also reduced substantially the risk of breast cancer recurrence in one large study.[13] Based on these data, 12 months of adjuvant trastuzumab is usually considered as the standard treatment for HER2-positive early breast cancer. Since adjuvant trastuzumab carries some risk of cardiac failure, cardiac monitoring at approximately 3-month intervals is either strongly recommended or considered mandatory during adjuvant trastuzumab treatment.

1.2 Rationale of the SOLD Study

Although administration of 12 months of adjuvant trastuzumab is currently generally considered as the standard, the optimal duration of trastuzumab administration is not known. The 12-month duration of trastuzumab administration was selected to be used in all major adjuvant trials, although this duration of administration is no better supported by research data than other time periods. Some investigators have speculated that prolonged

administration of trastuzumab might be beneficial and that trastuzumab might resemble in this aspect tamoxifen,[13] but there are currently little evidence indicating that the 12 month administration duration selected for most clinical trials is optimal. The HERA study[13] includes a 2-year administration arm, but the results are pending.

In line with the North American N9831 Trial data, the interim results of the FinHer trial suggest that concomitant administration of trastuzumab with a taxane is effective in the treatment of HER2-positive early breast cancer.[8] In the FinHer study trastuzumab was administered weekly concomitantly with either 3-weekly docetaxel or weekly vinorelbine, followed by 3 cycles of FEC in each arm. Docetaxel improved recurrence-free survival as compared to vinorelbine (hazard ratio 0.58, 95% CI 0.40 to 0.85), and trastuzumab improved recurrence-free survival as compared to the same chemotherapy administered without trastuzumab (hazard ratio 0.42, 95% CI 0.21 to 0.83). During a median follow-up time of 3 years these treatments were not associated with detectable cardiac toxicity. The data suggest that the combination of docetaxel plus 9-week concomitant trastuzumab is effective and well tolerated in the treatment of HER2-positive breast cancer. However, the subgroup of the study that evaluated adjuvant trastuzumab was small with 232 patients randomized to receive or not to receive trastuzumab.

The results of E2198 trial are important concerning the SOLD study, because the design of E2198 resembles that of SOLD. E2198 was a small randomized adjuvant trial that examined the cardiac effects of HP (trastuzumab [H] plus 3-weekly administered paclitaxel [P]) followed by AC (doxorubicin [A] plus cyclophosphamide [C]) among 234 breast cancer patients with HER2-positive (IHC 2+ or 3+) stage II disease.[14]

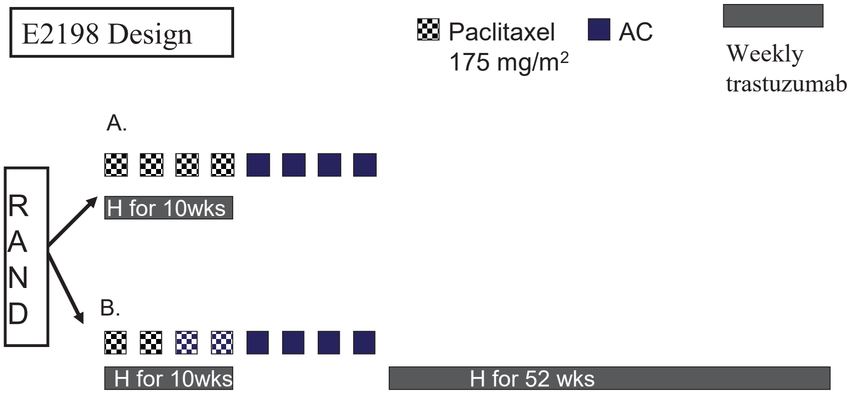

•234 HER2+ve (IHC 2+ or 3+) stage II BC

•Median FU 64 mo

•No difference in 5-yr DFS or OS

The patients were randomly allocated to receive either HP_{175} q3w X 4 followed by AC q3w x 4 (Arm A; HPx3→ACx4), or to the same regimen followed by H for 52 weeks (Arm B; HPx3→ACx4→H for 52 wks). The median follow-up at the time of reporting was 64 months. Disease-free survival at 5 years was equivalent for Arms A and B (76% vs. 73%, respectively, P= 0.55), and there was no difference in overall survival between the study arms (5-year survival was 88% for Arm A and 83% for Arm B, P=0.29). Seven study participants were diagnosed with congestive heart failure (Arm A, n=3; Arm B, n=4), all within 3 years from randomization. E2198 was not designed nor powered to test whether single-agent trastuzumab administered following chemotherapy influences survival, but the results of E2198 emphasize further the need to investigate the optimal duration of trastuzumab administration.

Hypothetically, all (or almost all) breast cancer cells need to be eradicated for cure. Concomitant administration of the most effective agents available is an obvious strategy to achieve complete eradication of all subclinical cancer. Based on in vitro data, the combination of trastuzumab and docetaxel may be one of the most synergistic ones of all the trastuzumab combinations available to date.[15] These in vitro findings are supported by clinical data suggesting high activity for the docetaxel plus trastuzumab regimen in the adjuvant,[8,14] preoperative systemic,[16,17] and metastatic settings.[18] Long duration of adjuvant administration of single-agent trastuzumab might also result in cancer cell eradication and gradual death of dormant cancer cell populations, although the bulk of evidence suggests that trastuzumab administered in combination with chemotherapy is more effective that trastuzumab given as a single agent.

Single-agent trastuzumab is generally well tolerated. Trastuzumab is not recommended to be administered in combination with anthracyclines (particularly doxorubicin) because of the risk of cardiac failure.[19] Due to the risk of cardiotoxicity cardiac monitoring is necessary when 12-month adjuvant trastuzumab is administered. Since administration of chemotherapy and subsequent single-agent trastuzumab lasts up to 18-months, many patient visits are required to complete the assigned treatment. Yet, similar results might be

achieved with shorter therapy that exploits drug synergism.[14,15] Short regimens might be associated with more limited toxicity, and the need to monitor the cardiac function might be avoided. If short regimens are associated with less cardiac toxicity, such treatments might be safer for patients with a moderately low LVEF, and such treatments might be preferred especially by breast cancer patients who have only a moderate risk of cancer recurrence. Short treatment regimens might also be an effective starting point for further development of novel adjuvant regimens.

In the present study 2 adjuvant regimens with different duration of trastuzumab administration are compared in the treatment of early HER2-positive breast cancer. Trastuzumab will be administered in both arms for 9 weeks in combination with docetaxel to exploit the putative synergism between these drugs and to ensure effective adjuvant therapy to all study participants regardless of the result of random allocation. All patients will also receive 3 cycles of anthracycline-containing chemotherapy, and those who have hormone receptor-positive breast cancer will be treated with adjuvant endocrine therapy for a minimum of 5 years.

2. STUDY OBJECTIVES

2.1 Primary Objective

The primary objective is to compare disease-free survival (DFS) of women treated with trastuzumab plus docetaxel (HT) followed by FEC to that of women treated with the same regimen followed by single-agent trastuzumab as the adjuvant treatments of early stage breast cancer.

2.2 Secondary Objectives

The secondary objectives include comparison of the treatment arms regarding

- Overall survival (OS)
- Distant disease-free survival
- Cardiac event-free DFS
- Left ventricle ejection fractions (LVEFs)
- Safety and toxicity of chemotherapy and trastuzumab, as assessed using the Common Terminology Criteria for Adverse Events (CTCAE) version 3.0
- and Quality Of Life (QOL)

3. STUDY DESIGN AND DURATION

3.1 Overview of Study Design

This is an open-label, 2-arm, prospective, randomized, multi-center phase III study. The study compares efficacy and safety of a taxane-trastuzumab-anthracycline chemotherapy regimen with the same regimen followed by single-agent trastuzumab as adjuvant treatments of women diagnosed with breast cancer with a high risk of cancer recurrence.

The study participants will be randomly allocated to one of the following 2 treatments in a 1:1 ratio:

A. Trastuzumab plus docetaxel (HT) (3 cycles) → 3-weekly $FE_{75}C$ (3 cycles) (HT→FEC)

B. Trastuzumab plus docetaxel (HT) (3 cycles) → 3-weekly $FE_{75}C$ (3 cycles) → 3-weekly trastuzumab to complete one year of trastuzumab administration (14 3-weekly cycles infusions; HT→FEC→T)

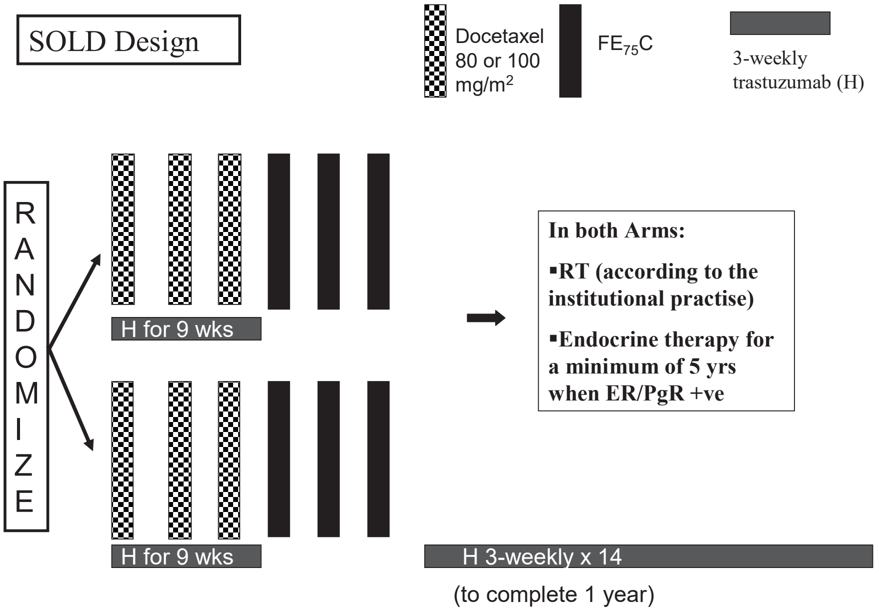

The study participants will be followed up for a minimum of 8 5 years post-randomization or until death.

Locoregional radiotherapy will be given according to the institutional practice. Radiotherapy can be given either before or after chemotherapy. When radiotherapy is given after chemotherapy, it is recommended to be started within 2 months after the last chemotherapy infusion (the third FEC).

Patients with ER and/or PgR positive disease will receive adjuvant endocrine therapy.

This is an international multi-center study with approximately 100 centers planning to participate.

3.2. Treatment Duration

Patients will receive 3 cycles (9 weeks) of trastuzumab plus docetaxel (HT) followed by 3 cycles (9 weeks) of CEF. In Arm B 14 cycles (42 weeks) of trastuzumab (H) will be administered following completion of chemotherapy. The total duration of trastuzumab administration is thus 9 weeks in Arm A and 51 weeks in Arm B.

Within 3 months of completing chemotherapy, adjuvant endocrine therapy will be initiated to all patients with ER and/or PgR positive disease, and endocrine therapy will be scheduled to be administered for a minimum of 5 years. The choice of adjuvant endocrine therapy is according to the institutional practice.

4. RANDOMIZATION PROCEDURE

Approximately 2,168 ~~3,000~~ patients will be enrolled in this study. Patients will be stratified by the axillary nodal status (pN0 or pN1 vs. pN2 or pN3), the HER2 analysis method (*in situ* hybridization or immunohistochemistry), ER expression (positive vs. negative) and the study center between the 2 treatment arms at the time of randomization, and allocated in a 1:1 ratio between the 2 treatment groups.

The HER2 status testing method (i.e. *in situ* hybridization or immunohistochemistry) will be reported during the randomization procedure.

Randomization will be carried out using a central computer-generated randomization system at the randomization center.

Table 1 – Information for Patient Randomization

Contact phone/fax numbers:	phone: to be provided<br>fax: +358 9 471 73 181
Study code name:	SOLD
Required information:	Patient's initials; month and year of birth<br>Center name and city<br>Axillary nodal status (pN0/pN1 or pN2/pN3)<br>Method of HER2 assessment (FISH/CISH or IHC)<br>ER status (positive or negative)<br>Date of obtaining informed consent

A unique Patient Study Number will be chronologically assigned by the central randomization center(s) as the patient is enrolled in the study. A subject may not be randomized to this study more than once. Randomization will be performed either by fax or via the world wide web.

5. STUDY POPULATION

5.1 Target Population

The intended population is female patients with invasive HER2-positive breast cancer with no overt distant metastases, and who are considered to have a high risk of breast cancer recurrence.

5.2 Inclusion Criteria

To be eligible for inclusion in the study, each patient must fulfill each of the criteria below:

- The patient must have provided a written informed consent prior to performing any of the study-specific procedures, with the understanding that she has the right to withdraw her consent at any time, without prejudice.
- Woman $\geq$ 18 years of age.
- Histologically confirmed invasive breast cancer.
- HER2-positive breast cancer (either FISH or CISH is positive; if these are not available immunohistochemistry is +++). HER2 expression is considered positive with immunohistochemistry when $\geq$80% of cancer cells are stained (corresponding to 3rd degree staining). If the HER2 status has been tested with both an *in situ* hybridization method and with immunohistochemistry, the *in situ* hybridization result will define the HER2 status of the tumor in case of discrepancy.
- The risk of breast cancer recurrence is considered high with either one of the following:
    - Pathological N0 (pN0) whenever the longest diameter of invasive cancer is >5 mm.
    - Histologically confirmed regional node-positive disease (pN+; either pN1mi, pN1, pN2, or pN3). Nodal isolated tumor cells/cell clusters (ITP) <0.2 mm in diameter are not considered as metastases.

5.3 Exclusion Criteria

Patients who fulfill any of the following criteria will be excluded:

- Presence of distant metastases.
- Inflammatory breast cancer.
- Pathological N0 with the longest tumor diameter from 6 to 10 mm *and* histological grade 1 (i.e. patients with pT1bN0M0 cancer are excluded from the study whenever histological grade is 1).
- Clinically significant (i.e. active) cardiac disease (e.g. congestive heart failure, symptomatic coronary artery disease and cardiac arrhythmia not well controlled with medication) or myocardial infarction within the last 12 months.
- Left ventricular ejection fraction less than 50% (or under the institutional normal reference range) assessed by echocardiography or isotope cardiography.
- ER, PgR, or HER2 status has not been determined.
- Primary systemic cancer therapy (neoadjuvant chemotherapy or endocrine therapy) has been administered prior to breast surgery.
- The World Health Organization (WHO) performance status > 1.
- Pregnant or lactating women.
- Women of childbearing potential unless using a reliable and appropriate contraceptive method. Women of childbearing potential (menstruating within 12 months of study entry), or with no hysterectomy and age < 55, must have a negative pregnancy test at baseline. Women amenorrheic for at least 12 months prior to study entry can be considered postmenopausal.
- More than 12 weeks between breast surgery and the date of randomization.
- Organ allografts with immunosuppressive therapy required.

- Major surgery (except breast surgery) within 4 weeks prior to study treatment initiation, or lack of complete recovery from the effects of major surgery.
- Participation in any investigational drug study within 4 weeks preceding study treatment initiation.
- Patients with a history of uncontrolled seizures, central nervous system disorders or psychiatric disability judged by the investigator to be clinically significant precluding study treatments.
- History of another malignancy within the last 5 years except cured basal cell carcinoma of the skin or carcinoma *in situ* of the uterine cervix.
- One or more of the following:
    - Blood hemoglobin $\leq$ 10.0 g/dL, neutrophils < 1.5 x 10^9/L, platelet count < 120 x 10^9/L
    - Serum/plasma creatinine > 1.5 x Upper Limit of Normal (ULN)
    - Serum/plasma bilirubin > ULN
    - Serum/plasma ALT (alanine aminotransferase) and/or AST (aspartate aminotransferase) > 1.5 x ULN
    - Serum/plasma alkaline phosphatase > 2.5 x ULN
- Serious uncontrolled infection or other serious uncontrolled concomitant disease (e.g. active peptic ulcer, unstable diabetes mellitus)
- Unwilling or unable to comply with the protocol for the duration of the study.
- History of hypersensitivity to the investigational products or to drugs with similar chemical structures.
- Pre-existing motor or sensory neurotoxicity of a severity ≥ grade 2 by CTCAE version 3, unless related to mechanical etiology.

6. THE SCREENING PROCEDURES

The screening procedures may be done in 2 stages. The first group of assessments can be done at any time within 28 days prior to randomization (day –28 to day 1). The second group (blood cell counts, blood biochemistry, and pregancy test for women with childbearing potential) must be done within 7 days prior to treatment start. If the assessments are performed on Day 1, the investigator must have reviewed the results prior to study drug administration. Refer to the Schedule of Assessments and Procedures (p. 9).

6.1 Informed consent, Eligibility Screening Form

A written informed consent must be obtained before any study-specific screening procedures are performed. The Eligibility Screening/Randomization Form (see the CRFs) is filled prior to randomization and faxed to the Study Administration Center or filled at the study web site.

6.2 Demographic Data

Demographic data includes age, race, the menopausal status and relevant surgical history (surgery for other cancers, vascular surgery, thoracic surgery).

6.3 Medical History

Medical history includes any major previous and concomitant diseases that may influence study drug administration or treatment outcome. Regularly taken concomitant medications and prior cancers will be listed on the CRFs.

6.4 Cancer / Treatment History

The assessment of cancer / treatment history includes:

- Date of first breast cancer histological diagnosis (the date of either core needle biopsy or excision biopsy)
- Type of breast surgery (mastectomy or breast conserving surgery)
- Method of assessing regional lymph nodes (sentinel node biopsy, evacuation of the axillary lymph nodes)
- Cancer histological type, histological grade (graded from 1 to 3), invasive tumor diameter, number of locoregional lymph node metastases (includes the axilla, ipsilateral parasternal nodes, supraclavicular fossa, intramammary nodes), the number of histologically examined lymph nodes
- UICC pTNM classification stage (International Union Against Cancer, TNM Classification of Malignant Tumours, 6th Edition)
- Hormone receptor status (ER and PgR, graded as positive or negative)
- Method of HER2 status evaluation (FISH/CISH or IHC)
- Representative tumor tissue block number/code

6.5 Physical Examination And Vital Signs

The general physical examination and assessment of vital signs includes:

- The World Heath Organization (WHO) Performance Status (see Appendix 1)
- Height (cm)
- Weight (kg)
- Pulse rate (beats/minute)
- Blood pressure (mm/Hg)

6.6 Laboratory Assessments

Normal ranges for the study laboratory parameters must be supplied to the Study Administration Center before entering patients to the study from the site.

The blood cell analyses assessed will include

- Haemoglobin content
- Leukocyte count
- Neutrophil count
- Platelet count

Blood biochemistry evaluated will include serum or plasma

- Creatinine
- Bilirubin
- ALT and /or AST

- Alkaline phosphatase.

Blood cell counts and blood biochemistry must be assessed within 7 days prior to the first study treatment (the first HT cycle). During the Chemotherapy Phase, the study laboratory tests must be performed within 3 days prior to the start of a new chemotherapy cycle and reviewed by the investigator before administration of study medications.

6.7 Pregnancy Test

Pregnancy test is required for women who are pre-menopausal or who are considered to be of childbearing potential, and for women who have been amenorrheic for less than 12 months.

Serum follicle stimulating hormone (FSH) will be measured whenever the patient's age is < 55 years and a hysterectomy has not been done to evaluate the menopausal status.

6.8 Left Ventricle Ejection Fraction (LVEF) and Electrocardiogram

The left ventricle ejection fraction of the heart is measured prior to the study (within 28 days prior to randomization), at the time of completion of chemotherapy (on Study Week 18 ± 2 weeks), on Study Week 31(±2 weeks), Week 43 (±2 weeks), Week 61 (±2 weeks) and on Study Month 36 (± 1 month). LVEF can be measured with either ultrasound (echocardiography) or with isotope cardiography (MUGA scan). The same evaluation method (either echocardiography or isotope cardiography) should be used throughout the study when evaluating any single study participant. The results are captured on the CRFs (the LVEF Assessment Form). The electrocardiogram (ECG) must be evaluated within 28 days prior to randomization.

6.9 Staging Examinations

Staging examinations are carried out according to the institutional practice. They are, however, mandatory when ≥ pN4+ (metastasis in 4 or more lymph nodes, pN2 or pN3), the primary tumor diameter is larger than 5 cm (pT3) and when the tumor is classified as pT4. These mandatory examinations include:

- chest CT *or* X-ray,
- isotope bone scan *or* skeletal X-ray (must include X-ray of the scull, the spine and the pelvis)
- CT, MRI, *or* ultrasound of the liver

At time of study entry, all staging studies must show no evidence of metastatic disease, including suspicious lymphadenopathy or skin nodules on physical exam. All other staging studies are at the treating physician's discretion. Any other staging test carried out (e.g., CT scans, MRI studies, ultrasound of the abdomen, PET scans not defined by the protocol) must also be negative for metastatic disease.

7. STUDY MEDICATION

7.1 Dose and Schedule of Study Medications

Study treatment must be initiated within 14 weeks as calculated from the latest surgery for breast cancer.

Table 2 - Study Medication Regimens

Arm	Drug	Dose	Treatment Days*
A and B	Trastuzumab ~~(Herceptin®)~~	Trastuzumab may be given either intravenously weekly or 3 weekly in combination with docetaxel, or subcutaneously 3-weekly in combination with docetaxel. ~~Each center must use the same trastuzumab administration schedule (either weekly or 3-weekly) throughout the study.~~ With IV trastuzumab, the first dose is given as 90-minute infusion, the subsequent doses as 30-minute infusions.	
		Weekly IV trastuzumab: First dose 4 mg/kg, subsequent doses 2 mg/kg; administered intravenously on Cycle Day 1, infused prior to docetaxel.	Weekly IV trastuzumab: Day 1, 8 and 15 of a 3-week cycle; for 3 cycles.
		3-weekly IV trastuzumab: The first dose is 8 mg/kg, the subsequent 2 doses 6 mg/kg; administered intravenously on Cycle Day 1, infused prior to docetaxel.	3-weekly IV trastuzumab: Day 1 of a 3-week cycle; for 3 cycles.
		3-weekly subcutaneous trastuzumab: Each dose is 600 mg regardless of the body weight or size. A volume of approximately 5 mL (with 10 000 U rHuPH-20) is injected usually into the thigh at a rate of over about 5 minutes.	3-weekly subcutaneous trastuzumab: Day 1 of a 3-week cycle; for 3 cycles.

	Docetaxel ~~(Taxotere®)~~	80 mg/m² or 100 mg/m² administered as 60-minute IV infusion (administered following completion of trastuzumab infusion)	On cycle Day 1, 3-weekly for 3 cycles
	Followed by		
	FEC	5-FU: 600 mg/m² IV<br>epirubicin: 75 mg/m² IV (≥60-minute infusion)<br>cyclophosphamide: 600 mg/m² IV	On cycle Day 1, 3-weekly for 3 cycles
B	Single-agent trastuzumab ~~(Herceptin®)~~	The first dose 8 mg/kg, the subsequent doses 6 mg/kg IV (first dose as 90-minute infusion, the subsequent doses as 30-minute infusions). Alternatively, s.c. trastuzumab 600 mg may be administered 3 weekly (the same dose regardless of the body weight, a volume of approximately 5 mL (with 10 000 U rHuPH-20) is injected usually into the thigh at a rate of over about 5 minutes).	On cycle Day 1, 3-weekly for 14 cycles

Upon completion of chemotherapy (HT/FEC) all patients who have ER +ve and/or PgR +ve disease will receive endocrine therapy for a minimum of 5 years. Adjuvant endocrine therapy should be initiated within 3 months of completing chemotherapy.

Locoregional radiotherapy will be given according to the institutional practice either prior to or after chemotherapy. When radiotherapy is after chemotherapy it is recommended to be started within 2 months of completing adjuvant chemotherapy (HT/FEC) (see 11., p. 40).

The doses of docetaxel, 5-fuorouracil, epirubicin, and cyclophosphamide are calculated per the body surface area, and that of trastuzumab per kilogram of the body weight (unless subcutaneous trastuzumab is used). Dose banding ±5% from the calculated exact dose is allowed.

7.2 Trastuzumab-Docetaxel (HT)

Trastuzumab must be administered first, followed by docetaxel.

Trastuzumab (H) administration

Three cycles of trastuzumab in total will be given concomitantly with docetaxel. Trastuzumab may be administered either weekly or 3-weekly in combination with

docetaxel. Each center is recommended to ~~must~~ use only one schedule of administration (either i.v. weekly or 3-weekly trastuzumab, or 3-weekly subcutaneous trastuzumab) throughout the study.

When weekly is chosen to be used, the starting dose (loading dose) is 4 mg/kg (Day 1), and all subsequent doses 2 mg/kg (Days 8 and 15 of Cycle 1, and Days 1, 8, and 15 of Cycle 2 and Cycle 3).

When 3-weekly trastuzumab is chosen to be used, the starting dose (loading dose) is 8 mg/kg (Day 1), and the subsequent doses 6 mg/kg, administered on Day 1 of the treatment cycle.

When subcutaneous trastuzumab is chosen to be used, a dose of 600 mg (a fixed dose regardless the body size) is administered on Day 1 of the treatment cycle.

Reloading of trastuzumab (i.e. in weekly administration 4 mg/kg instead of 2 mg/kg and in 3-weekly administration 8 mg/kg instead of 6 mg/kg) is recommended whenever an unscheduled break lasting longer than for 14 days occurs in trastuzumab plus docetaxel administration.Subcutaneous trastuzumab is always given at a fixed dose of 600 mg.

The duration of the first trastuzumab infusion is 90 minutes, and a minimum of 60 minutes is recommended be allowed to pass following completion of the trastuzumab infusion before starting docetaxel infusion. The patients are recommended to be carefully followed up for 6 hours after the first trastuzumab infusion, and at least for 2 hours after the subsequent infusions for allergic reactions. The second trastuzumab infusion may be administered intravenously over 30 minutes, and the docetaxel infusion is recommended to be started 30 minutes after completion of trastuzumab infusion. The third docetaxel infusion may be started immediately after completion of the trastuzumab infusion provided that no trastuzumab toxicity is detectable. Trastuzumab administration does not generally require any premedication.

Intravenous trastuzumab is obtainable commercially in vials containing 150 mg of trastuzumab. The contents of the vial is dissolved in sterile water as indicated in the label (other solvents, particularly glucose-containing solvents, should **not** be used). An appropriate volume of the resulting concentrate is infused in 250 mL of 0.9% sodium chloride solution intravenously (a glucose solution should not be used). Trastuzumab is compatible with commonly available administration sets including PVC sets.

Docetaxel (T) administration

Docetaxel 80 mg/m^2 or 100 mg/m^2 will be administered as a 60-minute intravenous infusion, on Day 1 of each 3-week cycle diluted in 250 mL of 5% glucose or in 0.9% sodium chloride. Three cycles in total will be given. Each participating center must define the starting dose of docetaxel and use it throughout the study. If the 80 mg/m^2 starting dose will be selected to be used, dose escalation to 100 mg/m^2 will not be allowed. Use of the starting dose of 100 mg/m^2 in the subset of patients who are younger than 60 at the time of randomization and the dose of 80 mg/m^2 when the subject is 60 or older at the time of randomization is allowed, provided that this practice is systematically followed and that the Coordinating Investigator is informed about this practice.

The docetaxel package insert states that all patients ought to be pretreated with oral corticosteroids to reduce the incidence and severity of fluid retention as well as the severity of hypersensitivity reactions. The recommended premedication is dexamethasone given as follows:

- 7.5 mg or 8 mg dose of dexamethasone the night prior to each docetaxel administration,
- 7.5 mg or 8 mg dose in the morning, one hour prior to the docetaxel administration,
- Subsequent 7.5 mg or 8 mg dose in the evening of the day of docetaxel administration,
- 7.5 mg or 8 mg dose in the morning and evening of the day after docetaxel administration,
- Final 7.5 mg or 8 mg dose in the second morning after docetaxel administration.

Antihistamines have not been shown to be useful in controlling of fluid retention associated with docetaxel administration. An equivalent corticosteroid to 8 mg of dexamethasone may be used (dexamethasone 8 mg = 8 mg betamethasone = methylprednisolone 40 mg = prednisone 50 mg = prednisolone 50 mg).

Docetaxel is obtainable commercially in vials of 80 mg (2.0 mL) and 20 mg (0.5 mL) and supplied with solvent. Once reconstituted with the solvent, the docetaxel premix solution has a concentration of 10 mg/mL. This should be further diluted with either 0.9% sodium chloride solution or 5% dextrose solution to produce a final concentration of 0.3 to 0.9 mg/mL, prior to administration. Docetaxel is compatible with commonly available administration sets including PVC sets.

7.3 Prophylactic G-CSFs Following Trastuzumab-Docetaxel Administration

Granulocyte colony stimulating factors (G-CSFs) may be used for primary prophylaxis or neutropenic infection following trastuzumab-docetaxel administration. The product to be used is at the discretion of the investigator. Suggested dosages and schedules are as follows:

- Filgrastim 300 µg or 480 µg subcutaneously daily for 5-7 days beginning on Day 2 after chemotherapy and ending at least 24 hours prior to the next cycle of chemotherapy

- Lenograstim 150 µg subcutaneously daily for 5-7 days beginning on Day 2 after chemotherapy and ending at least 24 hours prior to the next cycle of chemotherapy

- Pegfilgrastim 6 mg subcutaneously 24 hours following each cycle of chemotherapy (should not to be given later than 72 hours after chemotherapy administration)

7.4 5-Fluorouracil-Epirubicin-Cyclophosphamide (FEC)

FEC will be administered on Day 1 of every 3-week cycle. Three cycles in total will be given. The regimen consists of:

- 5-FU: 600 mg/m^2 via IV infusion
- epirubicin: 75 mg/m^2 via IV infusion (> 60 minute infusion)
- cyclophosphamide: 600 mg/m^2 via IV infusion

The recommended minimum duration of epirubicin infusion is 60 minutes to avoid high serum peak anthracycline concentrations.

7.5 Single-agent Trastuzumab (H) Following HT/FEC (Arm B)

Single-agent trastuzumab (H) is started 3 weeks (±1 week) after the date of administration of the last (the third) FEC cycle (on Week 19±1 of the study) for women allocated to Arm B. Trastuzumab is administered intravenously at 3-week intervals for 14 times (on Study Weeks 19, 22, 25, 28, 31, 34, 37, 40, 43, 47, 50, 53, 56, and 59). The first dose (administered on Week 19) is 8 mg/kg, and the subsequent doses (Weeks 22 to 59) 6 mg/kg. The first dose is administered over 90 minutes; the subsequent doses may be administered in 30 minutes. The patient is recommended to be followed up at least for 2 hours for allergic reactions following each infusion.

Alternatively, subcutaneous trastuzumab 600 mg may be administered 3 weekly (the same dose regardless of the body weight, in a volume of approximately 5 mL with 10 000 U rHuPH-20). Subcutaneous trastuzumab is injected usually into the thigh at a rate of over about 5 minutes). Subcutaneous trastuzumab is administered at 3-week intervals for 14 times (on Study Weeks 19, 22, 25, 28, 31, 34, 37, 40, 43, 47, 50, 53, 56, and 59).

Reloading of trastuzumab (i.e. administration of 8 mg/kg instead of 6 mg/kg) is recommended whenever an unscheduled break lasting longer than for 14 days occurs in single-agent trastuzumab administration.Subcutaneous trastuzumab is always given at a fixed dose of 600 mg.

7.6 Use of Prophylactic Antibiotics

Prophylactic use of antibiotics during the study chemotherapy phase is not mandated but is permitted at the discretion of the investigator. Prophylactic used of antibiotics is not necessary following single-agent trastuzumab administration ~~infusions~~.

7.7 Use of Prophylactic Antiemetics

Adequate control of nausea/vomiting is mandatory. The choice and duration of anti-nausea treatment is at the discretion of the investigator.

7.8 Determination of the Dose Based on BSA

In order to identify the appropriate dose of docetaxel, 5-FU, epirubicin and cyclophosphamide, perform the following steps:

- Measure the patient's actual height and weight to determine the body suface area (BSA)
- Derive the Body Surface Area using the nomogram found in Appendix 2.

Though the weight of the patient may change throughout the study, the surface area will be assumed to stay close to that measured at baseline. Dose adjustments for changes in body weight will be required when the change in weight is 10% or more.

Trastuzumab dosing will be based on weight. In case of a 10% or greater change in weight occurs as compared to the baseline, the trastuzumab dose must be adjusted accordingly. Subcutaneous trastuzumab is always given at a fixed dose of 600 mg.

7.9 Study Medication Administration Records

The clinic administration records must be kept for monitoring of compliance with therapy administered. The records must include

1. Date(s) of the drugs administered to the patient,
2. The quantity of the drugs administered to the patient.

The dates and quantities of chemotherapy agents and trastuzumab administered will be captured on the CRFs.

Endocrine therapy will not be monitored, but the type, quantity, and schedule of endocrine therapy will be captured on the CRFs.

The inventory must be available for inspection by the Clinical Trial Monitor.

8.0 CHEMOTHERAPY DOSE MODIFICATION FOR TOXICITY

8.1 Trastuzumab-Docetaxel (HT) Dose Modifications for Toxicity

The docetaxel dose will be reduced 20% (from 100 mg/m^2 to 80 mg/m^2, and from 80 mg/m^2 to 65 mg/m^2) in case one or more of the following occurs:

1. Febrile neutropenia (defined as fever of unknown origin without documented infection; $T_{ax} \geq 38.5$ and neutropenia with blood neutrophil count <1.0 x $10^9/L$)
2. Neutropenic infection (documented infection with blood neutrophils <1.0 x $10^9/L$)
3. Grade 4 neutropenia (blood neutrophil count <0.5x$10^9/L$) lasting longer than for 7 days (measurement of the nadir neutrophil counts are <u>not</u>, however, considered mandatory)
4. Any grade 3 or 4 non-haematological toxicity considered to be treatment-related by the investigator
5. Any treatment-related toxicity that results in hospitalization

If any of these toxicities recurs despite docetaxel dose reduction, the dose is further reduced 20% (from 80 mg/m^2 to 65 mg/m^2) for the third HT cycle.

Alternatively, docetaxel dose needs not to be reduced if toxicity is considered to result from neutropenia and G-CSFs will be administered during the remaining HT cycle(s). Doses below 65 mg/m^2 are not recommended to be used, and administration of G-CSFs should be considered.

No dose-reductions will be carried out for trastuzumab. Trastuzumab doses will not be reduced despite docetaxel dose reduction.

Treatment-related hematologic and non-hematologic adverse effects must have been resolved to grade ≤1 (except of alopecia and nail changes) as compared to the baseline prior administration of the next HT cycle. Neutropenia must have been resolved to grade 0 (>1.5 x $10^9/L$) prior to administration of the next HT cycle.

If administration of docetaxel needs to be deferred due to adverse effects or logistic reasons, trastuzumab administration will also be equally deferred.
Trastuzumab will thus always be administered on the same day prior to docetaxel.

The next cycle can be delayed up to for 21 days due to toxicity. If the patient has not recovered from the toxicity within 21 days as counted from the scheduled date of administration, administration of HT will be interrupted.

It is recommended that the HT cycles not given will be replaced by an equal number of $FE_{75}C$ cycles, followed by 3 further $FE_{75}C$ cycles. Trastuzumab is not administered concomitantly with FEC.

8.2 FEC Dose Modifications for Toxicity

The doses of 5-FU, epirubicin and cyclophosphamide will be reduced 17% to 20% (to 5-FU 500 mg/m^2, epirubicin to 60 mg/m^2, and cyclophosphamide to 500 mg/m^2) in case one or more of the following occurs:

1. Febrile neutropenia (defined as fever of unknown origin without documented infection; T_{ax} $\geq$38.5 and neutropenia with blood neutrophils <1.0 x 10^9/L)

2. Neutropenic infection (documented infection with blood neutrophils <1.0 x 10^9/L)

3. Grade 4 neutropenia (blood neutrophil count <0.5 x10^9/L) lasting longer than for 7 days (measurement of the nadir neutrophil counts are not, however, considered mandatory)

4. Any grade 3 or 4 non-haematological toxicity considered to be treatment-related by the investigator

5. Any treatment-related toxicity that results in hospitalization

If any of these toxicities recurs despite FEC dose reduction, the dose is further reduced 17% to 20% (5-FU to 400 mg/m^2, epirubicin to 50 mg/m^2, and cyclophosphamide to 400 mg/m^2) for the third FEC cycle.

Alternatively, 5-fluorouracil, epirubicin and cyclophosphamide doses need not be reduced if toxicity is considered to result from neutropenia and G-CSFs will be administered during the remaining FEC cycle(s).

Treatment-related hematologic and non-hematologic adverse effects must have been resolved to grade $\leq$1 (except of alopecia and nail changes) as compared to the baseline prior administration of the next FEC cycle. The next FEC cycle can, at the discretion of the treating physician, start once the neutrophil count has recovered to grade 1 (>1.0 x 10^9/L).

The next cycle can be delayed up to for 21 days due to toxicity. If the patient has not recovered from the toxicity within 21 days as counted from the scheduled date of administration, administration of FEC will be interrupted. The FEC cycles not given may be replaced by an equal number of CMF cycles at the investigator's discretion.

8.3 Single-Agent Trastuzumab (H) Dose Modifications for Toxicity (Arm B)

Whenever administration of trastuzumab has been discontinued due to trastuzumab-related toxicity during HT (e.g. anaphylactic reaction), single-agent trastuzumab will not be administered.

No dose reductions will be carried out. Administration of trastuzumab will be *discontinued* for any of the following reasons:

1. Any grade 3 or 4 non-haematological toxicity considered to be trastuzumab-related according to the investigator
2. Any grade 3 or 4 cardiac event
3. Symptomatic cardiac failure
4. Cardiac failure requiring medical management
5. Left ventricular ejection fraction (LVEF) decrease > 10 percentage points from the baseline value and to a value under 50% (e.g. from 60% to 48%; a decrease of 12 percentage points)
6. LVEF decrease to <45% from any baseline value (e.g. from 52% to 44%; a decrease of 8 percentage points)
7. Any toxicity considered to be trastuzumab-related that results in hospitalization

Patients whose LVEF decreases >15 percentage points from the baseline value to a value higher than 50% (e.g. from 68% to 52%; a decrease of 16 percentage points) will have trastuzumab administration *interrupted* for 4 weeks and LVEF reassessed. If no further decline in LVEF occurs, trastuzumab may be continued with close monitoring of the cardiac function.

Upon continuation of trastuzumab treatment a new loading dose of 8 mg/kg will be given whenever the unscheduled break lasts longer than for 14 days (unless subcutaneous trastuzumab is used, which is always given at the fixed dose of 600 mg), but the scheduled trastuzumab infusion(s) not given due to toxicity will not be replaced.

8.4 Special Notes Regarding Dose Modifications for Toxicity

- For any adverse event present at baseline, the dose modifications will apply according to the corresponding shift in toxicity grade, if the investigator feels this is appropriate. For example, if a patient has grade 1 sensory neuropathy at baseline that increases to grade 3 during treatment, this will be considered a shift of 2 grades and treated as a grade 2 toxicity for dose modification purposes.
- When several toxicities with different grades or severity occur at the same time, the dose modifications applied will be based on the most severe toxicity.
- For toxicities that are considered by the investigator unlikely to develop into serious events (e.g. alopecia, nail changes), treatment will be continued without dose reduction or dosing interruption. No dose reductions or interruptions will be required for anemia (unless hemolytic).

- If toxicity related to HT cycles requires a dosing delay or interruption for more than 3 weeks, the remaining chemotherapy cycles may be replaced by an equal number of FEC75 cycles at the discretion of the investigator.
- When docetaxel administration needs to be deferred due to toxicity, administration of trastuzumab is also deferred. Trastuzumab is always administered on the same day before docetaxel.

9. WARNINGS AND PRECAUTIONS

9.1 Trastuzumab

Cardiotoxicity. Trastuzumab is cardiotoxic and may cause decrease of the left ventricle ejection fraction (LVEF), and symptomatic cardiotoxicity including arrythmias and cardiac failure may occur. The LVEF of all study participants will be monitored either with echocardiography or isotope cardiography and compared to the baseline LVEF during Study Weeks 18 (±2 weeks), 31 (±2 weeks), 43 (±2 weeks), and Study Month 36 (±1 month) for patient safety. Should cardiac failure or a significant drop in the LVEF occur (see 8.3), trastuzumab administration must be discontinued. Most trastuzumab-related cardiac failures respond to conventional therapy (such as angiotensine converting enzyme inhibitors and diuretics). The long-term outcome of trastuzumab-related heart failure is not known.

Infusion-related or injection-related symptoms. Shivering and/or fever is often associated with the first trastuzumab administration ~~infusion~~. Other symptoms, such as nausea and vomiting, hypertension, pain, headache, cough, dizziness, rash, and lethargy may also occur, but these symptoms will usually diminish both in frequency and severity during subsequent infusions. Symptomatic medication, such as paracetamol, antihistamines, or pain medication may be required to control these symptoms.

Allergic reactions. Allergic reactions including anaphylactic reactions may occur with trastuzumab administration ~~infusion~~. Such reactions usually occur during the first administration ~~infusion~~ of trastuzumab, and they may be associated with hypotension, breathlessness, wheezing, hypertension, bronchospasm, tachycardia, anaphylaxis and shock, and they may (rarely) be fatal. Should such symptoms and/or signs develop, trastuzumab infusion must be discontinued, and the patient should be treated with appropriate therapy, such as oxygen administration, beta-agonists, corticosteroids, and epinephrine (adrenalin) at the treating physicians discretion. Patients with a history of severe hypersensitivity reactions should not be rechallenged with trastuzumab.

Contraindications: Trastuzumab is contraindicated in case of known hypersensitivity to trastuzumab or to mouse proteins.

Pregnancy: It is not known whether trastuzumab administration may harm the fetus if trastuzumab is administered to a pregnant woman. Use of trastuzumab should be avoided during pregnancy, unless the benefit to the mother is judged greater than the potential harm to the fetus.

9.2 Docetaxel

The incidence of treatment-related mortality associated with docetaxel therapy is increased in patients with abnormal liver function and in patients receiving higher doses. See Boxed Warnings of the docetaxel package insert.

Toxic deaths: Docetaxel administered at 100 mg/m^2 was associated with deaths considered possibly or probably related to treatment in 2.4% (34/1435) of patients with normal liver

function and in 11% (6/55) of patients with abnormal liver function (AST and/or ALT > 1.5 times UNL together with alkaline phosphatase > 2.5 times ULN). Among patients dosed at 60 mg/m^2, mortality related to treatment occurred in 0.6% (3/481) of patients with normal liver function, and in 3 of 7 patients with abnormal liver function. Approximately half of these deaths occurred during the first cycle. Sepsis accounted for the majority of the deaths.

Hepatic Impairment: (see Boxed Warning of the docetaxel package insert). Docetaxel should generally not be given to patients with bilirubin $\geq$ upper limit of normal (ULN), or to patients with AST and/or ALT $\geq$ 1.5 x UNL concomitant with alkaline phosphatase $\geq$ 2.5 x ULN.

Hypersensitivity reactions: Patients should be observed closely for hypersensitivity reactions, especially during the first and second infusions. Severe hypersensitivity reactions characterized by hypotension and/or bronchospasm, or generalized rash/erythema occurred in 0.9% of patients who received the recommended dexamethasone premedication. Should a hypersensitivity reaction occur, docetaxel infusion needs to be discontinued and appropriate therapy, such as oxygen and fluid administration, corticosteroids, and epinephrine (adrenalin), administered at the treating physicians discretion. Docetaxel must not be given to patients who have a history of severe hypersensitivity reactions to docetaxel or to other drugs formulated with polysorbate 80. Patients with a history of severe hypersensitivity reactions should not be rechallenged with docetaxel. See Boxed Warnings of the docetaxel package insert.

Hematologic Effects: Neutropenia (less than 1.0 x10^9/L) occurs in virtually all patients given 100 mg/m^2 or 80 mg/m^2 of docetaxel and grade 4 neutropenia (less than 0.5 x10^9/L) occurs in most patients given 100 mg/m^2 or 80 mg/m^2 of docetaxel unless leukocyte growth factors are administered. Frequent monitoring of blood cell counts is, therefore, essential so that dose can be adjusted. Docetaxel should not be administered to patients with a blood neutrophil count < 1.0 x10^9/L. Febrile neutropenia occurrs in about 10 to 25% of patients given 100 mg/m^2 of docetaxel without leukocyte growth factors.

Fluid Retention: Severe fluid retention occurs in approximately 5% of patients despite use of a 5-day dexamethasone premedication regimen in metastatic setting, when docetaxel is administered for longer periods of time than in the present protocol. It is characterized by one or more of the following events: poorly tolerated peripheral edema, generalized edema, pleural effusion requiring urgent drainage, dyspnea at rest, cardiac tamponade, or pronounced abdominal distention (due to ascites).

Pregnancy: Docetaxel can cause fetal harm when administered to pregnant women. Studies in both rats and rabbits at doses equal to or greater than 0.3 and 0.03 mg/kg/day, respectively (about 1/50 and 1/300 the daily maximum recommended human dose on a mg/m^2 basis), administered during the period of organogenesis, have shown that docetaxel is embryotoxic and fetotoxic (characterized by intrauterine mortality, increased resorption, reduced fetal weight, and fetal ossification delay). The doses indicated above also caused maternal toxicity. There are no adequate and well-controlled studies in pregnant women using docetaxel. If docetaxel is used during pregnancy, or if the patient becomes pregnant while receiving this drug, the patient should be informed of the potential hazard to the fetus or potential risk for loss of the pregnancy. Women of childbearing potential should be advised to avoid becoming pregnant during therapy with docetaxel.

10. MANAGEMENT OF TOXICITY

10.1 Neutropenia

Docetaxel and FEC administration is expected to be associated with neutropenia. Administration of docetaxel or FEC should **not** be interrupted if grade 4 neutropenia develops. The next HT treatment cycle can only start once the neutrophil count has recovered to grade 0 (>1.5 x 10^9/L). The next FEC cycle may be started once the neutrophil count has recovered to >1.0 x 10^9/L at the treating physician's discretion.

The platelet count should be >120 x10^9/L when the next HT or FEC cycle is initiated.

If neutrophil/platelet counts are below these values, re-administration is delayed for 3 to 7 days. If the counts improve over these limits, the treatment is re-administered at the previous dose level. However, if neutrophil count is still $\leq$ 1.5 x 10^9/L, and platelets < 120 x 10^9/L, the next cycle may be administered at a reduced dose.

Patients experiencing absolute neutrophil counts < 0.5 x 10^9/L (grade 4) longer than for 7 days or have febrile neutropenia (grade 3 or 4 neutropenia with fever ≥ 38.5°C) should have the dose of all chemotherapeutic agents reduced (for dose reduction, see 8.). Alternatively, G-CSFs may be administered. Patients who experience grade 3 or 4 neutropenia with a documented infection (clinical or microbiologic) follow the same guidelines as patients with febrile neutropenia.

10.2 Hypersensitivity Reactions

Patients who develop severe hypersensitivity reactions (hypotension with a blood pressure decrease of ≥ 20 mm Hg, bronchospasm, or generalized rash/erythema) should stop treatment immediately and be given appropriate therapy. These patients should not be rechallenged with the drug suspected to have caused the hypersensitivity.

10.3 Grade ≥ 2 Nausea/Vomiting

The administration of 5-HT3 antagonists is recommended for docetaxel-induced emesis and metoclopramide +/- 5-HT3 antagonists or cortocosteroids for FEC related nausea. Adequate prophylactic treatment has to be initiated once nausea or vomiting has occurred.

10.4 Fluid Retention

Severe (grade 3 or 4) fluid retention, possibly related to docetaxel, such as pleural effusion, pericardial effusion or ascites, should be closely monitored. In case of appearance of such toxicity, docetaxel dose should be reduced or treatment discontinued.

10.5 Hepatic Impairment

Serum bilirubin, ALT/AST and alkaline phosphatase concentrations should be measured and reviewed prior to each cycle.

Docetaxel should generally not be administered when serum bilirubin equals to or is above the ULN, or to patients who have ALT ≥ 1.5 x ULN concomitantly with alkaline phosphatase ≥ 2.5 x ULN. Such patients are at an increased risk for grade 4 neutropenia, febrile neutropenia, infections, thrombocytopenia, stomatitis, skin toxicity, and toxic death. Patients with isolated elevations of transaminase ≥ 1.5 x ULN also have a higher rate of grade 4 febrile neutropenias, and deferring of administration for a few days may be considered.

If the patient has serum bilirubin equal to or above the ULN, or AST or ALT ≥ 1.5 x ULN concomitantly with alkaline phosphatase ≥ 2.5 x ULN, docetaxel administration must be

delayed at least for one week. If the laboratory values have not recovered within 3 weeks (serum bilirubin below the ULN, and AST/ ALT < 1.5 x ULN), then docetaxel should be discontinued

11. RADIATION THERAPY

Radiation therapy to the ipsilateral breast, chest wall and the regional lymphatics will be administered according to institutional practice either prior to or after chemotherapy. When given after chemotherapy, radiotherapy is recommended to be started within 2 months after completing adjuvant chemotherapy as calculated from the date of administration of the last (the third) FEC. A guideline for radiotherapy administration is provided in Appendix 6.

Radiotherapy is considered mandatory following breast conserving surgery.

Radiation therapy should not be administered during chemotherapy (HT or FEC cycles) or within 2 weeks following the last FEC. Single-agent trastuzumab may be administered concomitantly during radiation therapy in Arm B.[20] The volume of the heart included within the target volume should be minimized in radiation therapy planning.

The target volume (breast/chest wall/axilla), and the total cumulative dose of radiation therapy administered will be captured on the CRFs (Radiation Therapy Form, CRF page 41).

12. ENDOCRINE THERAPY

Upon completion of chemotherapy, all patients who have ER positive and/or PgR positive disease will receive adjuvant endocrine therapy for a minimum of 5 years. The choice of the endocrine therapy administered is at the treating physician's discretion. Tamoxifen 20 mg/day, aromatase inhibitor (anastrozole, exemestane, letrozole) and LHRH agonists are all acceptable choices. A sequential use of tamoxifen and an aromatase inhibitor is also acceptable. Any form of ovarian function ablation (chemical, surgical or by irradiation) is considered an acceptable endocrine treatment for premenopausal women. The type and duration of endocrine therapy used will be captured on the CRFs (Endocrine Therapy Form).

Women who have been amenorrheic (without menstrual periods) longer than for a minimum of 12 months prior to randomization are considered postmenopausal. Serum follicle stimulating hormone (FSH) may be measured when the patient's age is <55 years and no hysterectomy has been done to evaluate the menopausal status.

The following endocrine treatment options are **not** allowed:

- Use of an aromatase inhibitor without concomitant ovarian function suppression in women who are premenopausal
- Experimental endocrine agents
- Agents not labeled for adjuvant treatment of early breast cancer

Based on concerns of increased risk of deep vein thrombosis and reduced efficacy of chemotherapy, endocrine treatment should not be given concurrently with chemotherapy. Endocrine treatment is recommended to be initiated within 3 months of completing chemotherapy.

Endocrine treatments should be administered at their recommended dosages and schedules. For tamoxifen the recommended dosage is 20 mg/day, for letrozole 2.5 mg/day,

anastrozole 1 mg/day, and exemestane 25 mg/day, all administered p.o once daily. Endocrine therapy may be administered concurrently with radiation therapy.

The institutional criteria for hormone receptor positive cancer may be followed, but all cancers with 10% or more positive cancer cells in immunohistochemistry need to be considered as receptor positive disease.

13. CONCOMITANT MEDICATION AND TREATMENT

13.1 Concomitant Medication

Patients may continue with their concomitant medications, as directed by their physician.

All regular concomitant medication used longer than for one month during the study will recorded on the CRFs (please see CRF page 49).

13.2 Major Surgery

Any major surgery (defined as surgery requiring general anesthesia or hospitalization longer than for 3 hours) should be recorded on the CRFs (please see CRF page 49).

13.3 Cardiac Treatment

All procedures performed to diagnose or treat cardiac disease should be recorded including the date, indication, and description of the procedure(s). Such procedures will be recorded on the CRFs (Cardiac Disease Reporting Form, CRF page 50).

13.4 Other Anticancer Therapies

The use of other cytotoxic agents, experimental drugs, breast cancer active or passive immunotherapy other than specified by the protocol is not allowed during the study.

Patients requiring radiotherapy during the study to other body sites than the breast and the regional lymphatics for treatment of cancer will be considered to have relapsed and should come off study before receipt of radiotherapy.

14. DISCONTINUATION OF THERAPY

Patients are expected to complete study treatment unless they experience intolerable toxicity, withdraw consent, or have cancer recurrence. Patients who experience cancer recurrence (distant metastases, locoregional relapse, or who are diagnosed with a new primary breast cancer or a second primary invasive cancer) will be off study, and will be treated according to the institutional practice.

15. PREMATURE WITHDRAWAL

Patients have the right to withdraw from the study at any time for any reason. The investigator has the right to withdraw a patient from the study due to adverse events, treatment failure, intercurrent illness, protocol violation, and for administrative reasons. Unnecessary withdrawal of patients must be avoided.

Following withdrawal the investigators should continue to record patient data as thoroughly as possible unless the patient withdraws her consent for such recording to occur. The investigator or research personnel should contact/meet the patient or a relative to determine the reason for the withdrawal. The specific event or test resulting in withdrawal will be recorded on the CRF.

After withdrawal, patients will be followed, if feasible, as the patients who continue to participate in the study.

16. SAFETY INSTRUCTIONS AND GUIDANCE

16.1 Clinical Adverse Events

An adverse event (AE) is defined as any untoward medical occurrence in a patient administered a pharmaceutical product and which does not necessarily have to have a causal relationship with this treatment. An adverse event can therefore be any unfavorable and unintended sign (including an abnormal laboratory finding), symptom, or disease temporally associated with the use of a medicinal product, whether or not considered related to the medicinal product. Pre-existing conditions that worsen during a study are to be reported as adverse events.

Adverse events will be graded based on the Common Terminology Criteria for Adverse Events (CTCAE) version 3 available at http://ctep.cancer.gov (Appendix 3) in the toxicity categories that have recommended grading. Adverse events that do not have a recommended grading will be graded according to the following intensity using a 4-point scale: mild, moderate, severe or life-threatening. Definition of the grades can be found below.

Table 3- The Intensity of Adverse Events not defined by CTCAEv.3.0

Mild or *Grade 1*	discomfort noticed but no disruption of normal daily activity
Moderate or *Grade 2*	discomfort sufficient to reduce or affect daily activity
Severe or *Grade 3*	inability to work or perform normal daily activity
Life Threatening or *Grade 4*	represents an immediate threat to life

Adverse events related to HT/FEC chemotherapy and/or trastuzumab administered will be captured on the CRFs.

Since it may be difficult to assess which adverse effects are related either to single-agent trastuzumab or to one of the concomitant therapies administered (such as endocrine therapy), Symptoms/Toxicity Forms will be filled for all study participants (regardless of the Arm and regardless of whether the patient receives trastuzumab or not) during the post-chemotherapy phase of the study (at visits on Weeks 19, 31, 43 and 61).

16.2 Laboratory Test Abnormalities

Laboratory test results will be recorded on the Symptoms/Toxicity Evaluation Forms of the CRFs during the Chemotherapy and Post-Chemotherapy phases of the study.

16.3 Expected Serious Adverse Reactions

The following grade 3 or 4 adverse effects are considered as unavoidable and expected adverse effects of the type of chemotherapy administered, and are **not** considered reportable serious adverse events:

- Grade 3 and 4 neutropenia within 3 weeks following chemotherapy administration
- Grade 3 and 4 leukopenia within 3 weeks following chemotherapy administration
- Grade 3 or 4 trombocytopenia within 3 weeks following chemotherapy administration

16.4 Suspected Unexpected Serious Adverse Reactions (SUSARs)

SUSARs are **serious, unexpected adverse reactions, considered to be related to study treatment that are not mentioned in the product label**. SUSARs must be reported by the investigator to the Sponsor within *one* working day of occurrence (expedited reporting). SUSARs are reported using the CIOMS Form (Appendix 5) or according to the national regulations. Any of the following outcomes/events need to be reported **within one working day using the CIOMS Form** to the Sponsor:

- Any adverse effect that is fatal (results in death)
- Is life-threatening (grade 4, except grade 4 neutropenia, leukopenia or thrombocytopenia); NOTE: the term "life-threatening" refers to an event in which the patient was at immediate risk of death at the time of the event; it does not refer to an event which could hypothetically have caused a death had it been more severe)
- Results in persistent or significant disability/incapacity
- Pregnancy during study treatment
- Congenital anomaly/birth defect
- Event leading to **unplanned** in-patient hospitalization or prolongation of in-patient hospitalization other than treatment for neutropenic fever or neutropenic infection/sepsis. Neutropenic infections are considered a common and expected result of modern cancer chemotherapy, and need not expedited reporting (these are reported using the 1-page study SAE Reporting Form, see below). Planned hospitalizations, such as hospitalization for a planned diagnostic procedure or for planned elective surgery, need not be reported.
- Other important medical events considered by the investigator to require expedited reporting

16.5 Serious Adverse Events (SAEs)

Serious adverse effects (SAEs) are severe (grade 3 or 4 according to CTCAEv3.0) adverse effects that are either expected results of chemotherapy or known adverse effects of the study drugs. The known SAEs are listed in the label of the drugs (please see the drug labels). **Known (expected) SAEs are excluded from the expedited reporting.** Such expected adverse events, although serious, such as grade 3 infections, stomatitis, diarrhea, skin rash, nausea, and fatigue, or febrile neutropenia, thus need not to be reported within one working day, nor is the CIOMS Form mandatory in reporting. However, in case of unexpected outcome of these events, the reaction should be reported expedited to the Sponsor.

The following adverse effects are considered as SAEs that need to be reported to the Sponsor using the 1-page Serious Adverse Event (SAE) Reporting Form (please see the CRF page 52) within 7 days after becoming to the investigator's attention:

- Any grade 3 non-haematological adverse effect
- Any grade 3 hematological finding other than grade 3 or 4 neutropenia, leukopenia or thrombocytopenia
- Any other grade 3 laboratory finding
- Hospitalization for treatment of neutropenic fever or neutropenic infection
- Cardiac failure, from any cause, that requires medication or intervention must be reported regardless of grade

SAEs will be reported during HT, FEC, and single-agent trastuzumab, and SUSARs during HT and single-agent trastuzumab, and for up to 28 days after the last intake of trastuzumab.

16.6 Treatment And Follow-up of Adverse Events

Adverse events will be recorded throughout study treatment and at least for 28 days after the last intake of a study drug (HT, FEC or trastuzumab). Severe, life-threatening or related events must be followed until resolution, the patient's death, or the relationship is re-assessed.

16.7 Pregnancy

In case of pregnancy, the investigator should counsel the patient regarding continuation of the study treatments, discuss the risks of continuing with the pregnancy and the possible effects on the fetus. Monitoring of the patient should continue until conclusion of the pregnancy.

The investigator must report all pregnancies within 24 hours the Sponsor using the CIOMS form. Pregnancies occurring up to 90 days after the completion of chemotherapy or trastuzumab administration must also be reported to the Sponsor.

.

17. ASSESSMENT OF QUALITY OF LIFE

Quality of life (QOL) will be measured using the 2-page EuroQol-5D instrument that is available in many languages (Appendix 4). QOL will be assessed at the following points in time **in both of the study Arms**:

- Within 28 days before initiation of chemotherapy (before the first HT cycle) (baseline QOL)
- On day 1 of the third HT cycle (study Week 7) within 48 hours prior to initiation of HT
- On day 1 of the third FEC cycle (study Week 16) within 48 hours prior to initiation of FEC
- On study Week 18 (±2 weeks), prior to initiation of single-agent trastuzumab in Arm B
- On study Week 31 (±2 weeks)
- On study Week 43 (±2 weeks)
- On study Week 61 (±2 weeks) (end-of study)
- On study Month 36 (±1 month)

The patient herself will fill the questionnaires and will return these to the study personnel after completion.

18. RESEARCH BLOOD / SERUM SAMPLES

A serum sample (2 mL) and plasma sample (2 mL) will be collected at time of screening, at end of treatment (Week 61 ±2 weeks) and annually during follow-up (Months 24, 36, 48, 60, 72 and 84). Collection of these samples is optional. The samples are stored at -20˚C or colder until shipment.

19. RESEARCH TISSUE SAMPLES

Tumor tissue samples, usually fixed with formalin and embedded in paraffin for long-term storage, may be collected for research and/or quality control purposes pending on obtaining the study participant's informed consent.

20. CORRELATIVE BIOLOGICAL STUDIES

Biological variables that may be associated with treatment outcome and/or resistance to treatment will assessed in exploratory studies. Therefore, serum, plasma, and tissue samples will be collected from the study participants (optional). The following analyses are planned to be performed from the samples collected:

1. Analysis of gene amplifications that may be associated with treatment efficacy, such as *TOP2A* and *c-MYC* amplifications. These analyses will be carried out from the primary breast cancer tissue.

2. Analysis of gene mutations that may be associated with treatment resistance, such as *PI3K* mutations.

3. Analysis of expression of the other HER family proteins and their downstream signaling molecules such as AKT, pTEN, and mTOR. These analyses are planned to be performed using a tissue microarray (TMA).

4. The serum/blood samples will be used for analysis of serum proteins or other soluble molecules that may or may not be associated with treatment outcome, such as soluble serum markers for cancer (e.g. HER2 ectodomain) or soluble growth factors to find out whether blood concentrations of such proteins can be used to identify the subset of patients who will benefit most (or least) from adjuvant anti-HER2 therapy. The serum/blood samples may also be used to study treatment-associated adverse effects (such as markers released from the cardiac muscle upon a chemical insult).

The tissue samples may be subjected to immunostaining of other proteins that may or may not be associated with treatment outcome (prognostic or predictive factors) or treatment-associated adverse effects.

21. HER2 REASSESSMENT FOR QUALITY CONTROL

Use of *in situ* hybridization techniques is recommended for *HER2* gene copy number analyses, but immunohistochemistry is also acceptable in assessing the HER2 status. The pathology laboratory performing HER2 status determination are recommended to be accredited to perform these analyses. Running a quality control program of HER2 status analysis is also strongly recommended.

Tumor tissue samples will be collected during the study for quality control purposes. Reanalysis of HER2 expression and ER/PgR expression will be carried out at one or more central laboratories from these samples. The central confirmation of the HER2 status needs not to be done prior to study entry.

22. STATISTICAL CONSIDERATIONS AND ANALYSIS PLAN

22.1 Primary and Secondary Study Variables

The primary variable of the study is comparison of disease-free survival (DFS) between the 2 treatment arms.

The secondary variables are overall survival (OS), distant disease-free survival, cardiac event-free DFS, change of left ventricle ejection fraction (LVEF) from the pretreatment value, frequency and severity of adverse events of therapy, and quality of life.

22.2 Analysis Populations

Intent-to-Treat population

All randomized patients will be included in the intent-to-treat population.

Per-Protocol population

The per-protocol population excludes randomized patients who did not receive at least one dose of trastuzumab and/or docetaxel, or who had a major violation of protocol inclusion or exclusion criteria.

Safety population

All patients who received at least one dose of trastuzumab and/or docetaxel will be included in the safety population. The safety population will be used for all analyses of safety.

22.3 Primary Efficacy Analysis (DFS)

The primary efficacy variable is the rate of disease-free survival.

Time to recurrence will be measured as the time from when the patient was randomized to the time she was first recorded as having disease recurrence (distant recurrence, locoregional recurrence, contralateral breast cancer, any invasive second cancer) or the date of death if the patient dies due to causes other than breast cancer recurrence. Patients without any such event or lost to follow-up will be censored at the time they were last confirmed not to have a recurrence.

The primary efficacy analysis will be done for the intent-to-treat population (all randomized patients). The results of this intent-to-treat analysis will be compared to the results of the per-protocol analysis (includes study participants who completed the assigned therapy according to the protocol). Possible inconsistencies in the results of the 2 analyses will be investigated and clarified in the study report.

Survival will be compared between the treatment arms using the Cox proportional-hazards model. In addition to a hazard ratio, an associated 95% confidence interval and a P-value calculated based on the Cox model, the results will be summarized by Kaplan-Meier plots, medians, and 95% confidence intervals for each treatment arm. To confirm the robustness of the statistical analysis, survival of the patients allocated to the 2 treatment arms will also be compared using the log-rank test.

The primary analysis will be performed when approximately ~~516~~ 366 events (recurrences or deaths) have been reached among the intent-to-treat population or when the last patient entered to the study has been followed up for 2.0 years after randomization (corresponding to a median follow-up time of approximately 5.75 years) ~~the median follow-up time exceeds 5 5.75 years~~, whichever will occur first. Also the results of all secondary endpoints shall be presented at that time.

Since chemotherapy administered is similar in both arms, no difference in DFS or OS can be expected to occur during the chemotherapy phase of the study. Therefore, as a sensitivity analysis DFS will also be evaluated between the study arms by comparing the events that occurred after the administration of the last FEC cycle (the 6th chemotherapy cycle) or after the 6th chemotherapy cycle administration, if FEC has been replaced by CMF. The patients who have an event before this timepoint will be considered as censored at the time of the event.

Another sensitivity analysis is planned to be carried out in the subset of patients who have centrally confirmed HER2-positive breast cancer. Confirmation of the HER2 status will be done from tissue blocks or tissue slides shipped for a second (confirmatory) analysis of the

HER2 status to a central laboratory. Patients whose cancer is deemed HER2 positive both by the local laboratory and a central laboratory will be included in the sensitivity analysis. Cases where the HER2 status has been determined in the local laboratory only will be excluded from the analysis.

22.4 Cardiac Event-free DFS

Cardiac event-free disease-free survival will be calculated from the date of randomization to the point in time (date) when the patient was first recorded as having one or more of the following events:

- cardiac heart failure (CHF) diagnosed after study entry and requiring medication or medical intervention
- myocardial infarction (any kind) diagnosed after study entry
- cardiac or coronary artery surgery, or coronary artery dilatation or stenting
- cancer recurrence (distant recurrence, locoregional recurrence, contralateral breast cancer, any invasive second cancer), or
- death from an intercurrent cause.

Patients who were not reported as having any of these events and patients who are lost to follow-up will be censored on the date they were last known to be alive without an event.

22.5 Distant Disease-free Survival And Overall Survival

Distant disease-free survival (DDFS) will be measured as the time from the date of randomization to the date of first diagnosis of distant recurrence of breast cancer or to the date of death from any cause. Cancer recurrences in the ipsilateral breast, in the regional lymph nodes, or in the chest wall/subcutaneous tissue/skin of the surgical bed are considered locoregional recurrences, and are not accounted for as events when DDFS is calculated. Patients who are alive without distant recurrence of breast cancer and patients who are lost to follow-up will be censored using the date they were last known to be alive.

Overall survival (OS) will be measured as the time from the date of randomization to the date of death. Patients who were not reported as having died at the time of the analysis and patients who are lost to follow-up will be censored using the date they were last known to be alive.

22.6 Adverse Events And Premature Withdrawals

Adverse effects considered to be related to the chemotherapy administered and/or to trastuzumab will be captured on the CRFs.

Adverse events will be reported as listings and summarized as frequency tables and will include summaries by severity. Laboratory data will be reported in the form of listings and frequency tables. Withdrawals of patients from study medication will be reported as listings and summary tables. All summaries and listings of adverse events and laboratory data will be based on the safety population.

22.7 LVEF

The left ventricle ejection fractions (LVEFs) will be compared between the treatment arms and to the baseline (prestudy) LVEFs using repeated measures analysis of covariance (RMANCOVA) model. The model will include the center/country, treatment arm, visit and

the interaction between the treatment arm and visit as fixed factors. The baseline LVEF will be used as a covariate. The overall difference over all post-baseline visits and visit-specific differences between the treatment arms will be estimated. If the assumptions of the RMANCOVA model are not fulfilled, corresponding non-parametric methods will be used. The means, medians and ranges of LVEFs will be summarized at the protocol-specified time points between the treatment arms. The proportions of patients who have over 15% reduction in the LVEFs, over 10% reduction to a value less than 50%, and of those who develop a LVEF smaller than 45% during the study will be tabulated. Frequency tables listing the numbers of patients who are diagnosed with cardiac failure or other cardiac toxicity will be produced. The numbers and proportions of patient who will have cardiac medication started or cardiac surgery/interventions will be reported per arm. Patients who are diagnosed with breast cancer recurrence or with a second cancer are censored from these analyses at the date of the diagnosis of cancer recurrence/second cancer.

22.8 Safety Analysis And Interim Analysis

Safety Analysis. It is important that therapy is not associated with substantial cardiac toxicity. Therefore, an interim safety analysis will be carried out when approximately 150 patients have completed study treatment with single-agent trastuzumab in Arm B. This interim safety analysis includes analysis of the LVEFs and other cardiac safety.

Safety analyses can be performed also at other times based on the recommendation of the independent Data Safety Monitoring Committee. Survival or cancer recurrence data will not be analyzed in these safety reviews.

~~**Planned Interim Analysis.** Toxicity of the treatments and early outcome data may be analyzed when a minimum of 1200 patients have been treated according to the protocol with HT, FEC, and single-agent trastuzumab (if allocated to Arm B), and followed up for a minimum of a median of 36 months. The tolerability of the treatments may be reported both at this time point and at the time of the final study report. If outcome data will be evaluated in the interim analysis, the method by O'Brien and Fleming will be used to adjust the significance level both in the interim analysis and in the final analysis.[21] If the two-sided p-value for the difference between the arms in DSF is less than 0.0052 in the interim analysis, it is possible to stop the study after the interim analysis. If the interim analysis will be conducted, a two-sided significance level less than 0.048 will be considered as statistically significant in the final analysis of DSF.~~

22.9 Sample Size Considerations (For revision, see 30.9 Appendix 9)

~~A superiority assumption is made between the study treatments. It is assumed that the survival rate after 5 years of follow-up will be 84.0% in the better arm and 80% in the worse arm (the treatments are similar for approximately 5 months [0.4 years] in the study arms). The number of required events is calculated using the formula by Collett[22] as follows:~~

$$e = \frac{4(z_{\alpha/2} + z_{\beta})^2}{\log(HR)^2},$$

~~where α is the 2-sided significance level, β is the power and HR is the hazard ratio between the treatment arms. Using a power of 0.80, a 2-sided significance level of 0.05 and a hazard ratio of 81 [log(84) / log(80)], altogether 516 events would be needed.~~

~~With these assumptions, approximately 1450 patients per each treatment arm would be needed. To account for patients who discontinue the study or are lost to follow-up (estimated 3%), a total of approximately 3000 patients will be enrolled to the study (1500 patients per arm). The sample size was calculated using the nQuery computer program version 6.0.~~

22.10 Replacement Policy

For patients

No patient who prematurely discontinued the study for treatments or was lost from follow-up any reason will be replaced.

For centers

A center may be closed and replaced for the following reasons:

- Excessively slow recruitment
- Poor protocol adherence.

23. STUDY DURATION AND DATES

The duration of this study is expected to be 114 ~~84~~ months, beginning with the first subject's first visit anticipated to occur in November 2007 and ending in May 2017 ~~November 2014~~ with the last subject's visit 2-year follow-up visit. The accrual is anticipated to last for 7.5 4 years.

24. GENERAL STUDY ADMINISTRATION

24.1 Study Steering Committee

The Study Steering Committee will include one or 2 representatives from any one country that will participate in conduction of the trial. In case of only one representative, a vice-representative needs to be nominated. Study Steering Committee may also include the study statistician, a representative of the Study Administration Center, and representative(s) of a major study Sponsor(s). The Coordinating Investigator must be a member of the Steering Committee.

24.2 Independent Data Safety Monitoring Committee

An Independent Data Safety Monitoring Committee with 3 or more members, not participating in the study, will be nominated. At least one of the members will be a professional cardiologist. The committee will assess the safety of study patients based on listings of adverse events, serious adverse events, and SUSARs. The committee will also review the process of the study and treatment efficacy.

25. ETHICAL ASPECTS AND PARTICIPANT PROTECTION

25.1 Declaration of Helsinki, GCP, And Local Regulations

The investigator must ensure that the study is conducted in accordance with the principles of the "Declaration of Helsinki" or with the laws and regulations of the country in which the research is conducted, whichever affords the greater protection to the individual. The study

must fully adhere to the principles outlined in “Guideline for Good Clinical Practice” (GCP) or with local law if it affords greater protection to the patient.

25.2 Patient Informed Consent

A written informed consent must be obtained from each patient participating in this study, after adequate explanation of the aims, methods, anticipated benefits, and potential hazards of the study, and prior to performing any of the study related procedures. For patients not qualified or incapable of giving legal consent, written consent must be obtained from the legally acceptable representative. The investigator must explain to the patients who consider participation in the study that they are completely free to refuse to enter the study or to withdraw their consent at any time, and for any reason. The informed patient consent will be included in the CRFs, and this must be completed appropriately. If new safety information results in significant changes in the risk/benefit assessment, the consent form will be updated if necessary. All participating patients should be informed of the new information, given a copy of the revised form and give their consent to continue in the study.

25.3 Institutional Review Board/Ethics Committee

The protocol, its modifications, and the consent forms will be reviewed and approved by an Institutional Review Board (Ethics Committee). This board must operate in accordance with the current national regulations. The investigator will send/fax a letter or certificate of protocol approval to the Study Administration Center prior to initiation of the study, and also whenever subsequent modifications to the protocol are made.

25.4 Conditions for Modifying the Protocol

Protocol modifications must be made only after consultation of the Study Steering Committee.

All protocol modifications that include changes in study treatments, inclusion or exclusion criteria, the numbers of patients randomized or any other changes that may influence patient safety, study conduction or study interpretation must be submitted to the appropriate Institutional Review Board for approval in accordance with local requirements, and to Regulatory Agencies if required. Approval must be awaited before any changes can be implemented, except for changes necessary to eliminate an immediate hazard to trial patients, or when the change(s) involves only logistical or administrative aspects of the trial (e.g. change in monitor(s), change of telephone number(s)).

25.5 Conditions for Terminating the Study

The Study Steering Committee reserves the right to terminate the study in case of poor accrual, or unexpected toxicity or outcome results. In terminating the study, the Steering Committee will consider protection of the patient’s interests.

26. STUDY DOCUMENTATION AND CRFS

26.1 Investigator's Files / Retention of Documents

The investigator must maintain accurate records for full documentation of the study and study data verification.

The Investigator's Study File will contain the protocol with amendments, CRFs, copies of the SAE reports, CIOMS forms, Institutional Review Board and regulatory agency approvals with correspondence, staff curriculum vitae and other appropriate documents.

Patient Clinical Source Documents include patient hospital/clinic records, physician's and nurse's notes, original laboratory reports, ECGs, X-ray/imaging results, and pathology reports.

The investigator must keep these documents on file for at least 15 years after completion or discontinuation of the study. After that period of time the documents may be destroyed, subject to local regulations.

Should the investigator wish to assign the study records to another party or move them to another location, the Study Administration Center must be notified.

26.2 Source Documents and Background Data

The investigator shall supply the Study Administration Center on request any required background data from the study documentation or clinic records. This may occur when CRFs are illegible or when errors are suspected. It is also necessary to have access to the complete study records when governmental queries occur or at requests for audit inspections. Patient confidentiality will be protected in such occasions.

26.3 Audits and inspections

Source documents for this trial must be made available to personnel monitoring and auditing the study, and to health authority inspectors after appropriate notification. The Case Report Forms must be made available for direct inspection by such parties and for comparison with the contents of the source documents.

26.4 Case Report Forms

The Case Report Form must be completed and signed by the investigator(s) or an authorized delegate from the study staff. This also applies to CRFs of those patients who discontinue the study. The reason for study discontinuation or withdrawal from the study must be noted on the CRFs (on the End of Trial Form, CRP page 50).

All forms must be typed or filled in using black ink, and must be legible. Errors should be crossed out but not obliterated, the correction inserted, and the change initialed and dated by the investigator or his/her authorized delegate. The investigator should ensure the accuracy, completeness, legibility, and timeliness of the data reported.

26.5 Confidentiality of Trial Documents And Patient Records

The investigator must assure maintaining of the study participants' anonymity at all occasions. Patients should not be identified a study identification code (randomization number; not by their name or the social security code). The investigator must keep a separate confidential patient enrollment log showing study randomization numbers and patients's identity codes/names.

27. MONITORING OF THE STUDY

Each study center will be monitored during the study at least once. At least the following items will be monitored at the monitor visits:

the randomization date; the allocation group; patient eligibility to the trial; starting dates of protocol-defined systemic treatments; end dates of protocol-defined systemic treatments, doses of protocol-defined systemic treatments administered; date of breast cancer recurrence, date and cause of death, dates of cardiac events, dates of cardiac surgery/stenting, date of second cancer, histology of second cancer, date of the last follow-up.

At least 50% of the CRFs must be monitored at every participating site and be compared with the source data to exclude any systematic error in data capturing and reporting. The monitor must have access to appropriate patient source data to verify the entries on the CRFs. It is recommended that the first monitoring visit should occur in the beginning of the study when only a few patients have been entered to the study.

The patient informed consents and correspondence with the Institutional Review Board and regulatory agencies will be monitored. Patient confidentiality will be maintained in accord with local requirements.

28. PUBLICATION OF DATA

The results of this study may be published as one or more publications and presented at scientific meetings. The final report will be published. The interim and early safety analysis reports may be published provided that the Study Steering Committee considers their publication to be valuable to the scientific community and/or to advance management of breast cancer patients.

Publication of a multicenter trial should only be done in entirety and not as individual center data. Both the Study Steering Committee and the Coordinating Investigator must agree with reporting of any results separately by single center(s).

The study reports will be authored by the investigators, including a study statistician. The directives of the International Committee of Medical Journal Editors will be respected when considering authorship (www.icmje.org).

29. REFERENCES

1. Polychemotherapy for early breast cancer: an overview of the randomised trials. Early Breast Cancer Trialists' Collaborative Group. Lancet 1998; 352:930-42.
2. Bonadonna G, Valagussa P, Moliterni A, Zambetti M, Brambilla C. Adjuvant cyclophosphamide, methotrexate, and fluorouracil in node-positive breast cancer: the results of 20 years of follow-up. N Engl J Med 1995: 332:901-6.
3. Pritchard-KI, Shepherd LE, O'Malley FP, et al. HER2 and responsiveness of breast cancer to adjuvant chemotherapy. N Engl J Med 2006;354:2103-11.
4. Knoop AS, Knudsen H, Balslev E, et al. retrospective analysis of topoisomerase IIa amplifications and deletions as predictive markers in primary breast cancer patients randomly assigned to cyclophosphamide, methotrexate, and fluorouracil or cyclophosphamide, epirubicin, and fluorouracil: Danish Breast Cancer Cooperative Group. J Clin Oncol 2005; 23:7483-90.

5. Howell A, Wardley AM. Overview of the impact of conventional systemic therapies on breast cancer. Endocr Relat Cancer. 2005; 12 Suppl 1:S9-16.
6. Effects of chemotherapy and hormonal therapy for early breast cancer on recurrence and 15-year survival: an overview of the randomised trials. Lancet 2005; 365:1687-717.
7. Slamon DJ, Clark GM, Wong SG, Levin WJ, Ullrich A, McGuire WL. Human breast cancer: correlation of relapse and survival with amplification of the HER-2/neu oncogene. Science 1987; 235:177-82.
8. Joensuu H, Kellokumpu-Lehtinen PL, Bono P, et al. Adjuvant docetaxel or vinorelbine with or without trastuzumab for breast cancer. N Engl J Med 2006;354:809-20.
9. Allred DC, Clark GM, Tandon AK, et al. HER-2/neu in node-negative breast cancer: prognostic significance of overexpression influenced by the presence of in situ carcinoma. J Clin Oncol 1992; 10:599-605.
10. Andrulis IL, Bull SB, Blackstein ME, et al. neu/erbB-2 amplification identifies a poor-prognosis group of women with node-negative breast cancer. Toronto Breast Cancer Study Group. J Clin Oncol 1998; 16:1340-9.
11. Joensuu H, Isola J, Lundin M, et al. Amplification of erbB2 and erbB2 expression are superior to estrogen receptor status as risk factors for distant recurrence in pT1N0M0 breast cancer: a nationwide population-based study. Clin Cancer Res 2003; 3:923-30.
12. Romond EH, Perez EA, Bryant J, et al. Trastuzumab plus adjuvant chemotherapy for operable HER2-positive breast cancer. N Engl J Med 2005; 353:1673-84.
13. Piccart-Gebhart MJ, Procter M, Leyland-Jones B, et al. Trastuzumab after adjuvant chemotherapy in HER2-positive breast cancer. N Engl J Med 2005; 353:1659-72.
14. Sledge GW, O'Neill A, Thor AD, et al. Adjuvant trastuzumab; Long-term results of E2198. Breast Cancer Res Treatm 2006; 100: Suppl1, p. S106.
15. Pegram MD, Lopez A, Konecny G, Slamon DJ. Trastuzumab and chemotherapeutics: drug interactions and synergies. Semin Oncol 2000; 6 Suppl 11:21-5.
16. Montemurro F, Choa G, Faggiuolo R, et al. A phase II study of three-weekly docetaxel and weekly trastuzumab in HER2-overexpressing advanced breast cancer. Oncology 2004; 66:38-44.
17. Coudert BP, Arnould L, Moreau L, et al. Pre-operative systemic (neio-adjuvant) therapy with trastuzumab and docetaxel for HER2-overexpressing stage II or stage III breast cancer: Results of a multicenter pahse II trial. Ann Oncol 2006; 17:409-14.
18. Marty M, Cognetti F, Maraninchi D, et al. Randomized phase II trial of the efficacy and safety of trastuzumab combined with docetaxel in patients with human epidermal growth factor receptor 2-positive metastatic breast cancer administered as first-line treatment: the M77001 study group. J Clin Oncol 2005; 23:4265-74.
19. Slamon DJ, Leyland-Jones B, Shak S, et al. Use of chemotherapy plus a monoclonal antibody against HER2 for metastatic breast cancer that overexpresses HER2. N Engl J Med 2001; 344:783-92.
20. Halyard MY, Pisansky TM, Solin LJ, et al. Adjuvant radiotherapy and trastuzumab in stage I-IIA breast cancer: Toxicity data from North Central Cancer Treatment Group Phase III trial N9831. Proc Am Soc Clin Oncol 2006; 24: 8s (abstract 523)
21. O'Brien, PC, Fleming TR. A multiple testing procedure for clinical trials. Biometrics 1979; 35:549-556.
22. Collett D. Modelling survival data in medical research. In Chapmann and Hall (Eds, 1994, Section 9.2)

30. APPENDICES

30.1 Appendix 1 - WHO Performance Status

Grade	Description
**0**	Able to carry out all normal activity without restriction
**1**	Restricted in physically strenuous activity but ambulatory and able to carry out light work
**2**	Ambulatory and capable of all self-care, but unable to carry out any work; up and about more than 50% of waking hours
**3**	Capable only of limited self-care, confined to bed or chair more than 50% of waking hours
**4**	Completely disabled; cannot carry out any self-care; totally confined to bed or chair
**5**	Dead

30.2 Appendix 2 - Nomogram for BSA Determination

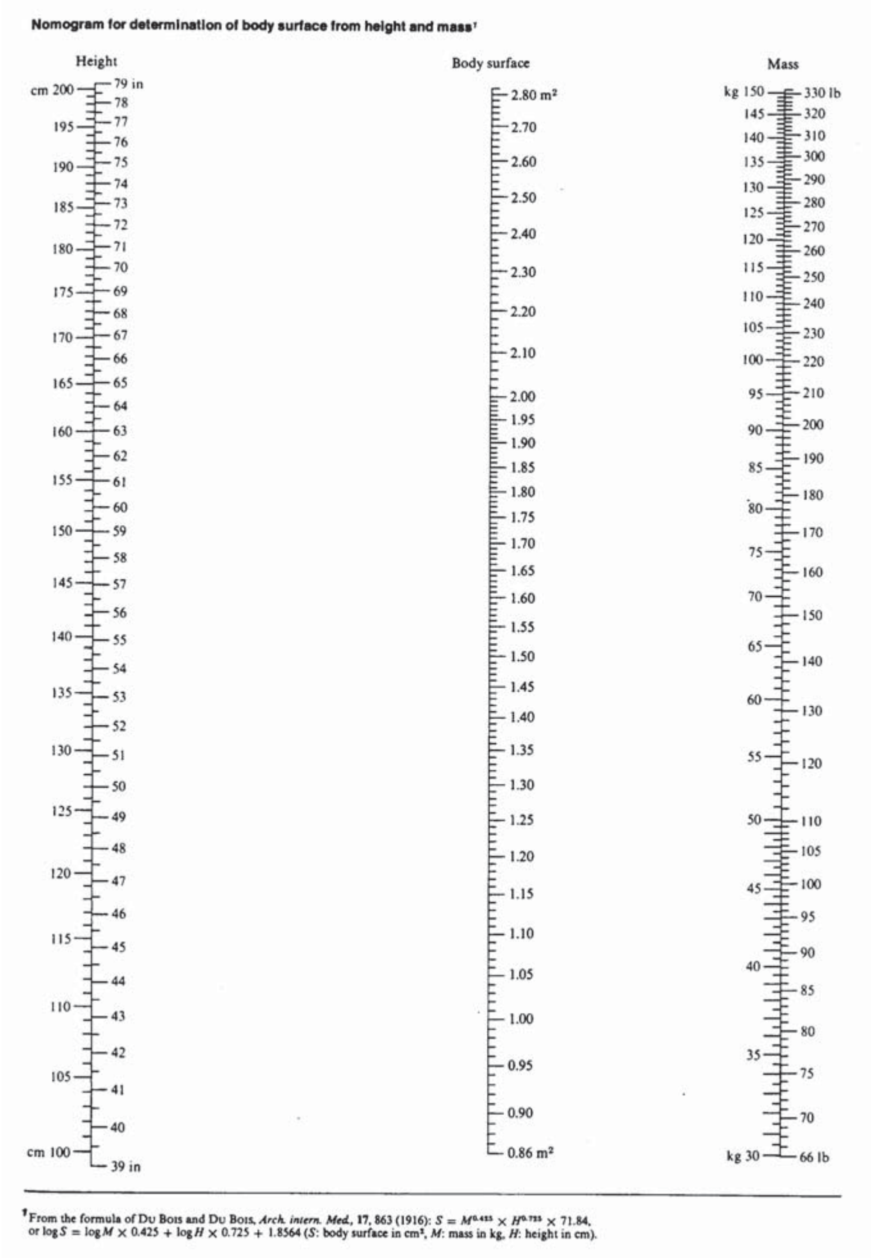

30.3 Appendix 3 - Common Terminology Criteria for Adverse Events v3.0 (CTCAE)

The Common Terminology Criteria for Adverse Events (CTCAE) version 3 can be found at the web site http://ctep.cancer.gov/reporting/ctc.html. Hard copies can be made available to sites through contacting the Sponsor.

30.4 Appendix 4 – EuroQol EQ-5D Quality of Life Instrument

For directions and examples of data analysis see http://www.euroqol.org/

Terveyskysely

(Suomalainen versio)

***(Finnish version*)**

Olkaa hyvä ja merkitkää rastilla (x), yksi rasti kunkin alla olevan ryhmän kohdalle, mikä väitteistä kuvaa parhaiten terveydentilaanne tänään:

Liikkuminen

Minulla ei ole vaikeuksia kävelemisessä ❑

Minulla on jonkin verran vaikeuksia kävelemisessä ❑

Olen vuoteenomana ❑

Itsestään huolehtiminen

Minulla ei ole vaikeuksia huolehtia itsestäni ❑

Minulla on jonkin verran vaikeuksia peseytyä tai pukeutua itse ❑

En kykene peseytymään tai pukeutumaan itse ❑

Tavanomaiset toiminnot *(esim. ansiotyö, opiskelu, kotityö, vapaa-ajan toiminnot)*

Minulla ei ole vaikeuksia suorittaa tavanomaisia toimintojani ❑

Minulla on jonkin verran vaikeuksia suorittaa tavanomaisia toimintojani ❑

En kykene suorittamaan tavanomaisia toimintojani ❑

Kivut/vaivat

Minulla ei ole kipuja tai vaivoja ❑

Minulla on kohtalaisia kipuja tai vaivoja ❑

Minulla on ankaria kipuja tai vaivoja ❑

Ahdistuneisuus/Masennus

En ole ahdistunut tai masentunut ❑

Olen melko ahdistunut tai masentunut ❑

Olen erittäin ahdistunut tai masentunut ❑

Auttaaksemme ihmisiä sanomaan, kuinka hyvä tai huono jokin terveydentila on, olemme piirtäneet lämpömittaria muistuttavan asteikon. Parasta terveydentilaa, jonka voitte kuvitella, merkitään siinä 100:lla ja huonointa 0:lla.

Haluaisimme Teidän osoittavan tällä asteikolla, miten hyvä tai huono Teidän terveytenne on mielestänne tänään. Olkaa hyvä ja tehkää tämä vetämällä alla olevasta laatikosta viiva siihen kohtaan asteikolle, joka osoittaa, miten hyvä tai huono terveydentilanne on tänään.

Terveydentilani tänään

Paras kuviteltavissa

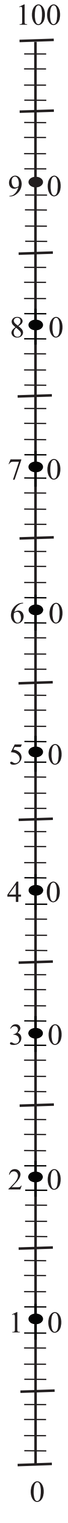

Huonoin

2. Hälsoenkät

3.

Svensk version

(Swedish version)

Markera, genom att kryssa i en ruta i varje nedanstående grupp (så här ☑), vilket påstående som bäst beskriver Ditt hälsotillstånd i dag.

Rörlighet

Jag går utan svårigheter ❑
Jag kan gå men med viss svårighet ❑
Jag är sängliggande ❑

Hygien

Jag behöver ingen hjälp med min dagliga hygien, mat eller påklädning ❑
Jag har vissa problem att tvätta eller klä mig själv ❑
Jag kan inte tvätta eller klä mig själv ❑

Huvudsakliga aktiviteter *(t ex arbete, studier, hushållssysslor, familje- och fritidsaktiviteter)*

Jag klarar av mina huvudsakliga aktiviteter ❑
Jag har vissa problem med att klara av mina huvudsakliga aktiviteter ❑
Jag klarar inte av mina huvudsakliga aktiviteter ❑

Smärtor/besvär

Jag har varken smärtor eller besvär ❑
Jag har måttliga smärtor eller besvär ❑
Jag har svåra smärtor eller besvär ❑

Oro/nedstämdhet

Jag är inte orolig eller nedstämd ❑
Jag är orolig eller nedstämd i viss utsträckning ❑

Jag är i högsta grad orolig eller nedstämd ❑

Till hjälp för att avgöra hur bra eller dåligt ett hälsotillstånd är, finns den termometer-liknande skalan till höger. På denna har Ditt bästa tänkbara hälsotillstånd markerats med 100 och Ditt sämsta tänkbara hälsotillstånd med 0.

Vi vill att Du på denna skala markerar hur bra eller dåligt Ditt hälsotillstånd är, som Du själv bedömer det. Gör detta genom att dra en linje från nedanstående ruta till den punkt på skalan som markerar hur bra eller dåligt Ditt nuvarande hälsotillstånd är.

Ditt nuvarande hälsotillstånd

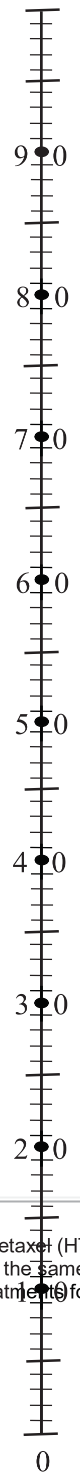

30.5 Appendix 5 – The CIOMS Form

CIOMS FORM

SUSPECT ADVERSE REACTION REPORT	A randomized phase III study comparing trastuzumab plus docetaxel (HT) followed by 5-FU, epirubicin, and cyclophosphamide (FEC) to the same regimen followed by single-agent trastuzumab as adjuvant treatments for early breast cancer<br>**SOLD**<br>(The Synergism Or Long Duration)<br>Protocol number FBCSG-01-2007<br>EudraCT number 2007-002016-26
Center:_____________________<br>Name, address, telephone number and fax number of investigator:____________	
Report type: ☐ Initial ☐ Follow up	

I. REACTION INFORMATION

	1a. COUNTRY:	2. DATE OF BIRTH			2a. AGE	3. SEX	4-6 REACTION ONSET			8-12 CHECK ALL
	PATIENT NUMBER:	Day	Month	Year	Years		Day	Month	Year	APPROPRIATE TO ADVERSE REACTION

7 + 13 SERIOUS ADVERSE EVENT(S) IN MEDICAL TERMS (use diagnosis if possible):<br><br>DESCRIBE REACTION(S) (including relevant tests/lab data) Serious Unexpected	☐ PATIENT DIED<br><br>☐ INVOLVED OR PROLONGED INPATIENT HOSPITALISATION<br><br>☐ INVOLVED PERSISTENT OR SIGNIFICANT DISABILITY OR INCAPACITY<br><br>☐ LIFE THREATENING

II. SUSPECT DRUG(S) INFORMATION

14. SUSPECT DRUG(S) (include generic name)		20. DID REACTION ABATE AFTER STOPPING DRUG?<br><br>☐ YES ☐ NO ☐ NA
15. DAILY DOSE(S)	16. ROUTE(S) OF ADMINISTRATION	21. DID REACTION REAPPEAR AFTER REINTRODUCTION?<br><br>☐ YES ☐ NO ☐ NA
17. INDICATION(S) FOR USE		
18. THERAPY DATES (from/to)	19. THERAPY DURATION	

III. CONCOMITANT DRUG(S) AND HISTORY

22. CONCOMITANT DRUG(S) AND DATES OF ADMINISTRATION (exclude those used to treat reaction)<br>Drug (substance) Begindate - Enddate
23. OTHER RELEVANT HISTORY (e.g. diagnoses, allergies, pregnancy with last menstrual period, etc.)
REPORTING DATE: ____<br><br>INVESTIGATOR'S SIGNATURE AND NAME IN BLOCK LETTERS:<br>____

30.6 Appendix 6 - A Guideline for Radiotherapy Administration

APPENDIX 6 RADIOTHERAPEUTIC PROCEDURES

General/Timing

Irradiation should be postponed until systemic treatment is completed. Ideally, it should commence 4 weeks after the last cycle of chemotherapy commences, However, it should start no later than 6 weeks after the last cycle of chemotherapy.

2 Radiotherapy indications

2.1 Chest wall radiotherapy

Chest wall radiotherapy following mastectomy should be considered for patients who fit any one of the following criteria [39]:

- **T3 tumours**
- **Four or more axillary nodes involved – mandatory:**
- **recommended if 1- 3 nodes involved**
- **Involved margins**

2.2 Radiotherapy to the breast itself

This is an integral part of any breast-conserving procedure and should be performed in all cases.

2.3 Nodal radiotherapy

Radiotherapy is recommended to include the axilla if an axillary sample or sentinel node biopsy has been positive and a full (usually level III) surgical clearance has not been performed. **In these cases, it is strongly recommended that a treatment technique is used which minimises any overlap, and that any match interface should not involve the axilla, a potential disease site.**

Radiotherapy to the axilla after a full level III axillary dissection must be avoided unless there is evidence of macroscopic residual disease in the axilla.

Irradiation of internal mammary nodes should be avoided so as to minimise the radiation dose to myocardium and lung.

Extracapsular spread in patients with involved axillary nodes does not constitute an absolute indication for axillary radiotherapy after surgical clearance of the axilla, given the higher risks of lymphoedema in these circumstances, and the lack of any evidence of survival benefit. Any treatment must only be considered after careful discussion with the patient on an individual patient basis.

Another controversial area is the case for a supraclavicular field in patients with more than three axillary nodes involved, especially, perhaps, those with apical node involvement: however radiation fields to include the supraclavicular fossa are strongly recommended in such cases.

Recommended technique

3.1 Position of the patient and simulation

The patients will be treated in the supine position, Acquirement of planning CT is recommended for radiotherapy planning and dosimetry. It is advised to assess the reproducibility by orthogonal laser beams.

3.2 Chest wall / Breast field.

Tangential fields will be used. Irradiation of large volumes of lung by the tangential fields should be avoided by keeping the central lung distance to less than 3 cm.

The chest wall may be irradiated using an electron beam of an appropriate energy (usually from 6 to 12 MV) from an anterior field.

For patients with left-sided tumours, the irradiation of large volumes of heart must be avoided by keeping the distance from the posterior edge of the field to the anterior border of the heart to <1.5 cm. If these parameters cannot be met, then we recommend that either full CT planning with voluming of the target and organs at risk, or the use of a lead cardiac shield on the medial field should be used.

If CT planning is not performed then a simulator film or digital image must be taken on the medial field to verify the above parameters have been met. A minimum of one transverse outline, taken on the central axis of the length of the tangential fields should be taken.

3.3 Axilla and supra-clavicular field.

Where the clinician feels these are a necessity, an anterior supraclavicular field with an opposed posterior axillary field will be used. The upper border will cover the supraclavicular fossa and is about 3 cm above the head of the clavicle. It is suggested that a gantry angle (usually of about 15%) is used to angle the field away from the spine. The medial border is the ipsilateral edge of the vertebral bodies. The lateral border should be placed at the insertion of Teres major onto the humerus. The lower field border should be matched onto the upper border of the tangential fields. If no chest-wall fields are to be used, then the lower border of the supra-clavicular field should be at the level of the

lower end of the head of the clavicle. The posterior axillary field should cover the apex of the axilla superiorly, the lower edge of anterior supraclavicular field inferiorly, and about to the lateral ends of the ribs medially. The use of a surgical clip is ideal to define the lower border of radiotherapy and upper border surgery, in the event of a level one clearance/sampling. Any shielding blocks will be indicated on a simulation film.

3.4 Supra-clavicular field.

Where the clinician feels this is a necessity, a single anterior field will be used. The infero-lateral corner should lie at the marker placed at the supra-medial limit of the axillary dissection. The upper border will cover the supraclavicular fossa and is about 3 cm above the head of the clavicle. It is suggested that a gantry angle (usually of about 15%) is used to angle the field away from the spine. The medial border is the ipsilateral edge of the vertebral bodies, the lateral border is guided by surgical clips if available, otherwise at the lateral extent of the second rib. The lower field border should be matched onto the upper border of the tangential fields. If no chest-wall fields are to be used then the lower border of the supra-clavicular field should be at the level of the lower end of the head of the clavical. Any shielding blocks will be indicated on a simulation film.

3.1.1 4. Dose and Fractionation

The dose distribution should be shown at least in the plane through the beam axes. The target area (PTV) in this plane should be outlined.

The tumour dose is specified at the reference point or iso-centre for the tangential fields, to the mid-plane for axillary fields and as an incident dose for the supraclavicular field.
A number of different dose/ fractionation schedules are in routine use. The following schedules are acceptable examples, to both the breast and nodal fields:

50 Gy / 25 daily fractions over 5 weeks
45 Gy / 20 daily fractions over 4 weeks
40 Gy / 15 daily fractions over 3 weeks

For patients having had conservative surgery, a boost to the tumour bed may be given in accordance with local protocol.

3.1.2 5. Treatment verification

Where local resources do allow, it is recommended that a weekly portal imaging film (or other recording when using on-line portal imaging systems) be obtained during the

course of treatment. Portal films should be compared to the simulator film. Field adjustments should be made in case of clinically important difference.

3.1.3 6. Alternative methods

Some centres have developed their own specific irradiation techniques for breast, chest wall, and supraclavicular treatments. Irradiation techniques and dosages differing markedly from those described in this recommendation can be allowed, provided a detailed description is given.

Alternative dose schedules are allowed if these are routinely employed by any centre, but the doses must remain constant for both arms of the trial and must be described in advance. The description of any markedly alternative techniques and/or dose/ fractionation schedules will be reviewed by the Steering Committee prior to inclusion as a trial participant.

30.7 Appendix 7 – Contact Addresses

Randomization Center

Clinical Trials Research Unit

Level 4, School of Population Health, Tamaki

The University of Auckland

Private Bag 92019, Auckland

Tel: +64 9 373 7599 ext 84742

Mobile: 027 6257301

Fax: +64 9 373 1710

www.ctru.auckland.ac.nz

Study Administration Center

The SOLD Study Center, Clinical Trials Unit, Department of Oncology, Helsinki University Central Hospital, Haartmaninkatu 4, FIN-00029 Helsinki
Phone: +358 9 471 75383
Fax: +358 9 471 73 181
email: mia.viskari@hus.fi

Study Sponsor

The Finnish Breast Cancer Group
Chair: Dr. Riikka Huovinen
Department of Oncology and Radiotherapy, Turku University Central Hospital,
Savitehtaankatu 1, FIN-20521 Turku, Finland
Phone: +358 2 313 0000
Fax: +358 2 313 2809
email: riikka.huovinen@tyks.fi

Coordinating Investigator

Prof. Heikki Joensuu
Department of Oncology, Helsinki University Central Hospital, Haartmaninkatu 4, FIN-00029 Helsinki
Phone: +358 9 471 3208
Fax: +358 9 471 74202
email: heikki.joensuu@hus.fi

30.8 Appendix 8 – Selection of aromatase Inhibitor for the study in Finland

Variations in medication decrease the possibility to detect a difference between the two study arms, and therefore it is recommended to keeping such variations to the minimum.

The Finnish Breast Cancer Group has selected letrozole (Femar®) as the aromatase inhibitor to be used in the study. The daily dose of letrozole is 2.5 mg, taken orally. Letrozole is not, however, considered as a study drug being investigated.

30.9 Appendix 9 – Amended power and sample size calculations (February, 2014)

The SOLD (Synergism Or Long Duration) is a randomised phase III trial examining the effect of trastuzumab plus docetaxel (HT) followed by 5-FU, epirubicin and cyclophosphamide (FEC) to the same regimen followed by single-agent trastuzumab given up to a total duration of 12 months as adjuvant treatments for early HER2-positive breast cancer. At present (February 2014) 12 months is considered the standard duration of adjuvant trastuzumab treatment (based on, for example, a unanimous decision of the St. Gallen expert consensus panel; Goldhirsch A et al. Ann Oncol 2013; 24:2206-23). Therefore, it is reasonable to consider the 12-month trastuzumab arm of SOLD as the standard arm and the 9-week arm as the experimental arm. Cardiac toxicity has been identified as the main toxicity of trastuzumab. Clinically significant cardiac toxicity has been reported to occur infrequently (Goldhirsch A et al. Lancet 2013: 382:1021-28; Perez EA. Clin Breast Cancer 2008; 8 Suppl 3:S114-20).

The SOLD protocol is based on a superiority design regarding the main endpoint (DFS). The sample calculation was carried out considering a 4% difference in DFS between the groups, 80% vs. 84% after 5-years of follow-up, using a power of 0.80, a 2-sided significance level of 0.05 and a hazard ratio of 0.781. With these assumptions, 516 events would be needed for the analysis. 3% of the patients were estimated to be lost to follow-up or to discontinue the study. A sample size of 3000 patients (1500/group) was estimated to be needed based on these assumptions.

These study power calculations need to be revised for the following reasons:

1) Recent data suggest that the assumptions made for 5-year DFS may be too low (Perez et al. J Clin Oncol 2011; 29:3366-3373; Perez et al. J Clin Oncol 29:4491-

4497, Goldhirch et al. Lancet 2013; 382:1021-28; Gianni et al. Lancet Oncol 2011; 12:236-44).

2) It may be unreasonable to assume that the experimental arm could be superior to the standard arm in terms of DFS, since deaths from trastuzumab-related toxicity are likely few, and the shorter treatment is not likely to reduce the number of breast cancer recurrences more than the longer treatment. Therefore, a non-inferiority design (with 1-sided testing) seems a more reasonable approach than a superiority design.

Coincidentally, patient accrual has been slower than anticipated. The first patient was entered to the study in January 2008, and accrual was anticipated to be completed 4 years later in January 2012. At present (February 21, 2014) 1969 patients have been randomised. Since the accrual rate has been approximately 300 patients per year, completion of accrual (up to 3000 randomised patients) is anticipated to take approximately 3 to 4 further years, at which time accrual would have lasted for a total of approximately 9 years. The longer than expected accrual period does affect the power calculations.

The best available evidence to inform sample size estimations is probably the NCCTG N9831 trial patient population (Perez et al. J Clin Oncol 2011; 29:4491-97). In this study women with early HER2-positive breast cancers were treated with an anthracycline-taxane chemotherapy regimen as in SOLD and unlike in HERA, where most patients did not receive taxanes. In N9831 some of the study participants were assigned to trastuzumab given concomitantly with a taxane. This is likely relevant, as concomitant administration of trastuzumab with chemotherapy (a taxane) is likely more effective than sequential administration of chemotherapy and trastuzumab (Perez et al. J Clin Oncol 2011; 29:4491-97). Such a regimen was used in arm C of the N9831 trial. Furthermore, survival data after a median of 6 years of follow-up is available from the N9831 trial (Perez et al. J Clin Oncol 2011; 29:4491-97).

In arm C of the N9831 trial 5-year DFS was 84.4%, based on 949 randomised patients and 313 events. It should be noted that only 13.9% of the patients assigned to arm C of N9831 had node-negative cancer. This proportion will likely be larger in SOLD, and therefore 5-year DFS is likely higher than 84.4% in the 12-month arm of SOLD.

Based on these data, 5-year DFS is estimated to be approximately 85.0% in the 12-month arm of SOLD. To study whether the 9-week regimen is non-inferior to the 12-month regimen, more than a 4% decrease in absolute DFS at 5-years is considered clinically relevant and is selected as the noninferiority margin. This absolute limit (4%) is maintained from the previous power calculations and a difference smaller than this is thus considered not significant from a clinical point of view. This margin translates to a 30% maximum relative detriment of 30% in DFS between the treatments, i.e. a relative non-inferiority margin (delta value) of 1.3 in this setting (i.e. the comparator baseline 5-year DFS of 85%). In other words, it is considered that a relative difference in 5-year DFS between the 9-week and 12-month arms of less than 30% does not produce effects that are clinically significant – and this results in absolute 5-year DFS differences of less than 4% not being clinically significant when based on an 85.0% 5-year DFS expected for 12-month treatment.

Another issue that influences the estimated sample size is whether testing is done 1-sided or 2-sided (using the alpha level of 0.05). 1-sided testing is often recommended in non-inferiority trials (e.g. Rothmann M. et al. Stat Med 2003; 22:239-64), as manifested in 1-sided testing using a 5% alpha level being the most common choice in non-inferiority trials as reported in a recent review of randomised studies carried out in breast cancer and lung cancer (Saad ED, Buyse M. Acta Oncol 2012; 51:890-6).

The table below shows the associations between the estimated DFS and the absolute and relative difference non-inferiority margins, and the effect of using 1-sided or 2-sided testing on the sample size:

**Estimated 5-year DFS rate (%) (irrespective of the duration of adjuvant trastuzumab)**	**Non-inferiority margin**		**Total Sample size 1-sided**	**Total Sample size 2-sided**	**Required number of events (1- / 2-sided)**
	**Absolute (%)**	**Relative**			
88.0	4	1.364	1822	2314	257 / 326
86.1	4	1.318	2006	2546	324 / 412
85.0	4	1.297	2104	2670	366 / 464
84.4	4	1.287	2154	2734	388 / 493
84.0	4	1.280	2196	2786	406 / 515

82.1	4	1.253	2370	3010	486 / 617

Assuming 7.5-year accrual, 2-year follow-up after completion of accrual, 80% power and a 95% (one- or two-sided) confidence interval.

Based on the current recruitment status of the study the duration of the recruitment period is estimated to be 7.5 years. Using 1-sided testing approximately 2104 patients need to be accrued, if the relative non-inferiority margin is set at 1.3 where the baseline 5-year DFS is 85.0% (hence an absolute non-inferiority margin of 4.0%), hence 366 events using 1-sided testing. Assuming a 3% drop-out rate the final sample size of the study is 2168 patients (1084 patients per group). This sample size will provide a power of approximately 80%. This sample size estimation is based on an assumption that the study cohort will be followed-up for a minimum of 24 months after entering the last patient of the cohort to the study.

A randomized phase III study comparing trastuzumab plus docetaxel (HT) followed by 5-FU, epirubicin, and cyclophosphamide (FEC) to the same regimen followed by single-agent trastuzumab as adjuvant treatments for early breast cancer

The Synergism Or Long Duration (SOLD) Study

Study code: SOLD

Phase III study

STATISTICAL ANALYSIS PLAN

APPROVED: Feb 28, 2017

Signatures:

Statistical Analysis Plan was prepared by:

________________________________ ______________________

Teppo Huttunen
Study Statistician | Date

Statistical Analysis Plan was reviewed/approved by:

________________________________ ______________________

Jouni Junnila
Senior Statistician | Date

Statistical Analysis Plan was reviewed/approved by:

________________________________ ______________________

Heikki Joensuu
Principal Investigator | Date

Table of Contents

1 STUDY OBJECTIVE(S) 74

2 DESIGN AND TYPE OF THE STUDY 74

3 SAMPLE SIZE CONSIDERATIONS 75

4 STATISTICAL HYPOTHESES 75

5 ANALYSIS SETS 76

5.1 Intention-to-treat (ITT) set 76
5.2 Per protocol (PP) set 76
5.3 Safety set 76

6 GENERAL STATISTICAL CONSIDERATIONS 76

6.1 On the non-inferiority limit 76

7 DEMOGRAPHIC AND OTHER BASELINE CHARACTERISTICS 76

8 ANALYSIS OF EFFICACY 77

8.1 Primary efficacy variable 77
8.2 Secondary efficacy variables 77
8.3 Additional analyses 78

9 ANALYSIS OF SAFETY 79

9.1 LVEF 79
9.2 Adverse events 79
9.3 Premature withdrawals 79
9.4 Other safety variables 79

10 OTHER VARIABLES 79

11 EXECUTION OF STATISTICAL ANALYSES 80

12 HARDWARE AND SOFTWARE 80

13 REFERENCES 80

14 APPENDICES 80

4. STUDY OBJECTIVE(S)

The primary objective is to compare disease-free survival (DFS) of women treated with trastuzumab plus docetaxel (HT) followed by 5-fluorouracil-epirubicin-cyclophosphamide (FEC) to that of women treated with the same regimen followed by single-agent trastuzumab as the adjuvant treatments of early stage breast cancer.

5. DESIGN AND TYPE OF THE STUDY

This is an open-label, 2-arm, prospective, randomized, multi-center phase III study. The study compares efficacy and safety of a taxane-trastuzumab-anthracycline chemotherapy regimen with the same regimen followed by single-agent trastuzumab as adjuvant treatments of women diagnosed with breast cancer with a high risk of cancer recurrence.

The study participants were randomly allocated to one of the following 2 treatments in a 1:1 ratio:

A. Trastuzumab plus docetaxel (HT) (3 cycles) → 3-weekly $FE_{75}C$ (3 cycles) (HT→FEC)

B. Trastuzumab plus docetaxel (HT) (3 cycles) → 3-weekly $FE_{75}C$ (3 cycles) → 3-weekly trastuzumab to complete one year of trastuzumab administration (14 3-weekly cycles; HT→FEC→T)

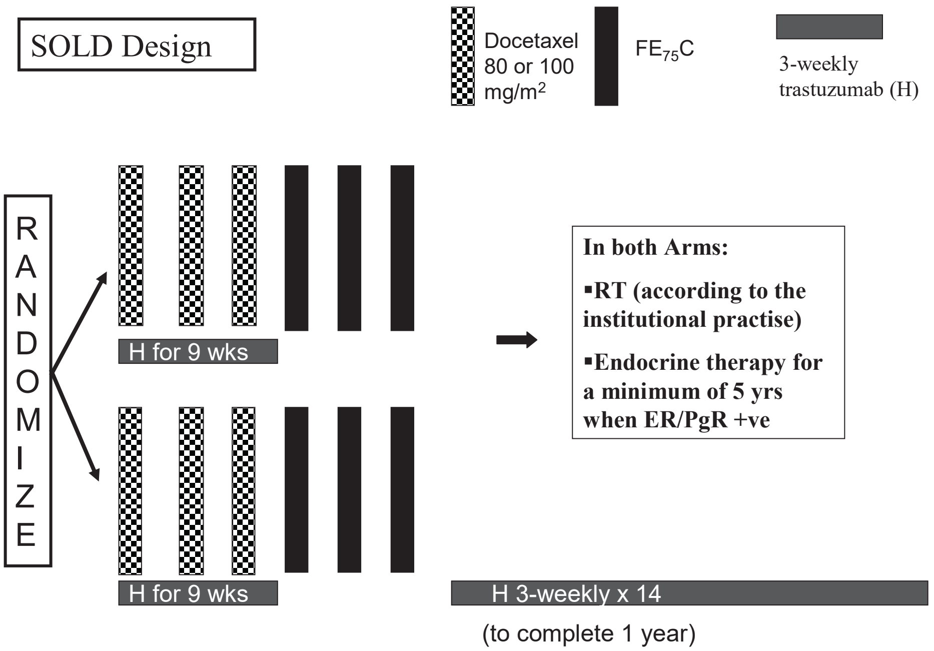

The study participants were to be followed up after the study has completed accrual for a minimum of 2 years post-randomization or until death to achieve a median follow-up time of 5 years.

Locoregional radiotherapy was given according to the institutional practice. Radiotherapy can be given either before or after chemotherapy. When radiotherapy was given after chemotherapy, it was recommended to be started within 2 months after the last chemotherapy infusion (the third FEC).

Patients with ER and/or PgR positive disease will receive adjuvant endocrine therapy.

This is an international multicenter study with a total of 63 participating centers in 7 countries (Finland, Sweden, UK, Belgium, New Zealand, Serbia and Iceland).

6. Sample size considerations

Original sample size of the study was planned to be 3000 patients (1500 per group). The sample size was amended during the study after it became evident that the patient accrual time will be substantially longer than anticipated. At this time, it was decided that a non-inferiority approach will be used in the primary comparison in place of the superiority approach, since it seemed unlikely that the shorter regimen could result in fewer DFS events as compared with the 12-month administration of trastuzumab given the relatively low toxicity of adjuvant trastuzumab. Futhermore, data from other trials had accumulated suggesting that the DFS estimates used in the power calculations were too low (Perez et al. J Clin Oncol 2011; 29:4491-97, see the updated sample size calculation document for Final Amended protocol dated 21Feb2014[1]).

The sample size amendment was based on the following calculations. Using 1-sided testing applied with N-I approach 2104 patients need to be accrued to achieve 366 events, which is needed if the relative non-inferiority margin is set at 1.3. Assuming a 3% drop-out rate the final sample size of the study is 2168 patients (1,084 patients per group). This sample size will provide a power of approximately 80%.

This was based on the assumption of 85.0% DFS in the standard treatment arm (arm B: trastuzumab until one year) and the non-inferiority margin of 4.0%, corresponding to relative margin of 1.3. Following table illustrates the connection of absolute and relative difference.

Table 1: Absolute vs relative N-I margins

Estimated 5-year DFS rate (%) (irrespective of the duration of adjuvant trastuzumab)	Non-inferiority margin		Total Sample size 1-sided	Total Sample size 2-sided	Required number of events (1- / 2-sided)
	Absolute (%)	Relative			
90.0	4	1.431	1700	2158	193 / 244
88.0	4	1.364	1822	2314	257 / 326
86.1	4	1.318	2006	2546	324 / 412
85.0	4	1.297	2104	2670	366 / 464
84.4	4	1.287	2154	2734	388 / 493
84.0	4	1.280	2196	2786	406 / 515
82.1	4	1.253	2370	3010	486 / 617

Note: Assuming 7.5-year accrual, 2-year follow-up after completion of accrual, 80% power and a 95% (one- or two-sided) confidence interval.

The accrual into the study was stopped after 2176 patients (after approximately 7 years of accrual, from the beginning of 2008 to the end of 2014), which is the final sample size of the study. As the Table 1 shows, an 80% power can be achieved already with approximately 200 events if the standard DFS rate is around 90%. At least this number of events was considered to be reached based on the accumulating data when the data cut-off date was set to the end of the year 2016 (December 31, 2016), i.e. fulfilling the 2-year minimum follow-up.

7. Statistical hypotheses

The purpose of this study is to investigate non-inferiority of the experimental treatment arm A (trastuzumab plus docetaxel (HT) (3 cycles) → 3-weekly $FE_{75}C$ (3 cycles)) when compared to the standard treatment arm B (trastuzumab plus docetaxel (HT) (3 cycles) → 3-weekly $FE_{75}C$ (3 cycles) → 3-weekly trastuzumab to

complete one year of trastuzumab administration). A hazard ratio (HR) between the treatment groups will be calculated for Disease Free Survival (DFS), and the null-hypothesis to be tested is

H_0 : treatment arm A is not non-inferior to treatment arm B as measured by a hazard ratio for the probability of disease recurrence over time (arm A / arm B)

against the alternative hypothesis

H_1 : treatment arm A is non-inferior to treatment arm B as measured by a hazard ratio for the probability of disease recurrence over time (arm A / arm B)

A one-sided 95 % CI for the hazard ratio (arm A / arm B) is used in the evaluation of the hypothesis. Non-inferiority is shown if the upper limit of the 95 % CI is less or equal to hazard ratio corresponding to a 4% absolute difference (see table 1 for the conversions).

8. Analysis sets

The analysis set based on the intent-to-treat (ITT) principle will be the primary dataset for the efficacy analysis. Patient classification for the datasets will be done prior to conducting the primary analysis.

Intention-to-treat (ITT) population

All randomized, appropriately consented patients will be included in the ITT dataset.

Per protocol (PP) population

The per-protocol population analysis (a modified Intention-to-Treat analysis) excludes randomized patients who did not receive at least one dose of trastuzumab and/or docetaxel, or who had a major violation of protocol inclusion or exclusion criteria. Lack of signed informed consent, absence of breast cancer, and presence of overt distant metastases on the date of randomisation are considered major violations of the protocol inclusion or exclusion criteria, and, therefore, such patients are excluded from the per protocol-analysis.

Safety set

All patients who received at least one dose of trastuzumab and/or docetaxel will be included in the safety population. The safety population will be used for all analyses of safety.

9. General statistical considerations

Primary analysis of non-inferiority will be conducted based on the 1-sided 95% confidence limit. To be consistent, the secondary survival analyses will also be done using same principle. In the statistical analyses a p-value less than 0.05 will be considered as statistically significant. If not stated otherwise, all other statistical tests will be performed as two-sided tests. No adjustments for the p-values will be made.

On the non-inferiority limit

Using meta-analysis results for (1-year trastuzumab vs standard chemo, DFS)[2] and a 50% retention approach[3] N-I limits ranging around 1.3 where obtained (up to 1.4 if single studies with results before option to cross-over are available[4]). Thus, they were in line with what is presented in the sample size considerations in chapter 3. See an appendix about NI generated at the time of protocol amendment.

10. Demographic and other baseline characteristics

The comparability of the baseline data will be checked by calculating descriptive statistics (mean, median, standard deviation, minimum and maximum for the continuous variables and frequencies and percentages for

categorical variables). In the ITT population, experimental treatment arm A (trastuzumab plus docetaxel (HT) (3 cycles) → 3-weekly $FE_{75}C$ (3 cycles)) will be compared to the standard treatment arm B (trastuzumab plus docetaxel (HT) (3 cycles) → 3-weekly $FE_{75}C$ (3 cycles) → 3-weekly trastuzumab to complete one year administration). In addition to the descriptive statistics, formal statistical methods will be used to flag potential differences in the baseline data between the treatment groups, if feasible. For continuous variables, Mann-Whitney U test will be used. For categorical variables, chi-squared test will be used. Cancer/treatment history will be summarised with tabulations and listings as appropriate.

11. ANALYSIS OF EFFICACY

Primary efficacy variable

The primary efficacy variable is Disease Free Survival (DFS) which will be measured as the time from when the patient was randomized to the time she was first recorded as having disease recurrence (distant recurrence, locoregional recurrence, contralateral breast cancer, any invasive second cancer) or the date of death if the patient dies due to causes other than breast cancer recurrence. Patients without any such an event or lost to follow-up will be censored at the time they were last confirmed not to have recurrence.

The primary analysis between groups will be conducted using the Cox proportional-hazards model and an associated one-sided 95% confidence interval to a hazard ratio (treatment arm A/treatment arm B) will be calculated to test the non-inferiority hypothesis. The primary comparison will be done using the ITT population (all randomized patients). The results of this intent-to-treat analysis will be compared to the results of the per-protocol analysis. The primary model will include the stratification factors used in the randomisation (axillary nodal status: pN0/pN1 or pN2/pN3, and ER status: positive or negative) as covariates. Randomisation was also stratified by center, but the center will not be included in the primary model due to the small size of many centers. An unadjusted analysis will also be conducted and its results be compared to the primary model.

In addition, the survival curves will be compared between the experimental (treatment arm A) and standard (treatment arm B) groups with use of the Kaplan-Meier plots, medians, and 95% confidence intervals for each treatment arm.

Since chemotherapy administered is similar in both arms, no difference in DFS (or OS) can be expected to occur during the chemotherapy phase of the study. Therefore, as a sensitivity analysis DFS will also be evaluated between the study arms by comparing the events that occurred after the administration of the last FEC cycle (the 6th chemotherapy cycle) or after the 6th chemotherapy cycle administration, if FEC was replaced by CMF. The patients who have an event before this time point will be excluded from this analysis.

Secondary efficacy variables

The following secondary variables will be analyzed similarly as the primary efficacy variable:

- Overall survival (OS)
- Distant disease-free survival (DDFS)
- Cardiac disease-free survival (Cardiac DFS)

Overall survival (OS) will be measured as the time from the date of randomization to the date of death. Patients who were not reported as having died at the time of the analysis and patients who are lost to follow-up will be censored on the date they were last known to be alive.

Distant disease-free survival (DDFS) will be measured as the time from the date of randomization to the date of first diagnosis of distant recurrence of breast cancer or to the date of death from any cause. Cancer recurrences in the ipsilateral breast, in the regional lymph nodes, or in the chest wall/subcutaneous tissue/skin of the surgical bed are considered locoregional recurrences, and are not accounted for as events when DDFS is calculated. Patients who are alive without distant recurrence of breast cancer and patients who are lost to follow-up will be censored on the date they were last confirmed not to have recurrence.

Cardiac disease-free survival (Cardiac DFS) will be calculated from the date of randomization to the point in time (date) when the patient was first recorded as having one or more of the following events:

- cardiac heart failure (CHF) diagnosed after study entry and requiring medication or medical intervention
- myocardial infarction (any kind) diagnosed after study entry
- cardiac or coronary artery surgery, or coronary artery dilatation or stenting
- cancer recurrence (distant recurrence, locoregional recurrence, contralateral breast cancer, any invasive second cancer), or
- death from an intercurrent cause

Patients who were not reported as having any of these events and patients who are lost to follow-up will be censored on the date they were last known to be alive without an event.

Additional analyses

Additional endpoints to be compared between treatment groups include:

- proportions of patients who developed brain metastases
- proportions of patients who developed second cancers (other cancer than breast cancer)

All survival endpoints are planned to be studied in the following subgroups:

- largest centers (over 100 patients) vs. other centers
- Axillary nodal status: pN0/pN1 or pN2/pN3
- Axillary nodal status: pN0 or pN+
- Number of histologically confirmed axillary nodal metastases: 0, 1-3, or >3
- Stage I (pT1N0)
- Stage 1B (breast tumor size 6-10 mm, pN0)
- Stage IC (breast tumor size 11-20 mm, pN0)
- Stage II (breast tumor size 50 mm or smaller and pN0; or breast tumor size 20 mm or smaller and 1-3 histologically confirmed axillary nodal metastases)
- ER status: positive or negative
- docetaxel dose: 80 mg of docetaxel per square meter vs. 100 mg of docetaxel per square meter
- By country
- Age (at randomisation), using a cut-off value of 50 years or younger

The subgroup analyses will be performed by including the subgroup factor in the Cox proportional-hazards model as a covariate (and testing the interaction effect with the treatment group). In addition, the Kaplan-Meier life-table method will be used to illustrate the effects in the subgroups. For completeness, the primary comparison between the treatments will be done using a 2-sided log-rank test.

12. ANALYSIS OF SAFETY

All patients who received at least one dose of trastuzumab and/or docetaxel will be included in the safety population. The safety population will be used for all analyses of safety.

LVEF

The left ventricle ejection fractions (LVEFs) will be compared between the treatment arms and to the baseline (prestudy) LVEFs using a repeated measures analysis of covariance (RMANCOVA) model. The model will include the treatment arm, visit and the interaction between the treatment arm and visit as fixed factors. The baseline LVEF will be used as a covariate. The overall difference over all post-baseline visits and visit-specific differences between the treatment arms will be estimated. If the assumptions of the RMANCOVA model are not fulfilled, corresponding non-parametric methods will be used. The means, medians and ranges of LVEFs will be summarized at the protocol-specified time points between the treatment arms. The proportions of patients who have over 15% reduction in the LVEFs, over 10% reduction to a value less than 50%, and of those who develop a LVEF smaller than 45% during the study will be tabulated. Frequency tables listing the numbers of patients who are diagnosed with cardiac failure or other cardiac toxicity will be produced. The numbers and proportions of patient who will have cardiac medication started or cardiac surgery/interventions will be reported per arm. Patients who are diagnosed with breast cancer recurrence or with a second cancer are censored from these analyses on the date of the diagnosis of cancer recurrence/second cancer.

Adverse events

Adverse events (classified by CTC) considered to be related to the chemotherapy administered and/or to trastuzumab will be reported as listings and summarized as frequency tables by the treatment group and will include summaries by severity. SAEs will be summarised separately by the treatment group.

Premature withdrawals

Withdrawals of patients from study medication will be reported as listings and summary tables by the treatment group including the reasons for discontinuation.

Other safety variables

Vital signs will be summarised by the treatment groups using descriptive statistics including changes from the baseline. Information about chemotherapy cycles, trastuzumab administration, radiation and endocrine therapy will be summarised by the treatment group with tabulations and listings.

13. OTHER VARIABLES

The quality of life, measured with the EQ-5D questionnaire, will be summarised by the treatment group using descriptive statistics. If feasible, the sum score will be tested between groups using analysis of variance models. Other information captured on the CRFs will be reported using listings and also other summaries when needed.

14. EXECUTION OF STATISTICAL ANALYSES

The final statistical analyses will be performed by 4Pharma Oy (study statistician Teppo Huttunen).

Statistical analyses of the SOLD trial will not be initiated before this document has been signed by the study statistician(s) and the trial Coordinating Investigator.

15. HARDWARE AND SOFTWARE

Statistical analysis will be performed with SAS® version 9.3 or later for Windows (SAS Institute Inc., Cary, NC, USA).

16. REFERENCES

1. Study protocol Final amended version dated Feb 21st 2014.
2. Rothmann M, Li N, Chen G, Chi GYH, Temple R, Tsou HH. Design and analysis of non-inferiority mortality trials in oncology. Statist. Med. 2003; 22:239–264. 2003.
3. Wenjin Y, Yiwei J, Zhenzhou S, Zhimin S, Jinsong L. Trastuzumab in the Adjuvant Treatment of HER2-Positive Early Breast Cancer Patients: A Meta-Analysis of Published Randomized Controlled Trials. PLoS ONE 6(6): e21030. 2011.
4. Trastuzumab after Adjuvant Chemotherapy in HER2-Positive Breast Cancer
Martine J. Piccart-Gebhart, M.D., Ph.D. et al, for the Herceptin Adjuvant (HERA) Trial Study Team N Engl J Med 2005; 353:1659-1672

17. APPENDICES

Appendix 1 Memo: On the computation of non-inferiority limit (18.7.2014)
