# Supplementary material for: Long-Term Outcomes of Adjuvant Trastuzumab for 9 Weeks or 1 Year for ERBB2-Positive Breast Cancer: A Secondary Analysis of the SOLD Randomized Clinical Trial
Source: JAMA Netw Open. 2024 Aug 26;7(8):e2429772. doi: 10.1001/jamanetworkopen.2024.29772 (PMC12507463; doi:10.1001/jamanetworkopen.2024.29772)
Supplement: Supplement 2. — eTable 1. Multivariable Cox Proportional Hazards Regression Model for Disease-Free Survival eTable 2. Multivariable Cox Proportional Hazards Regression Model for Overall Survival eFigure 1. CONSORT Diagram of the Trial eFigure 2. Disease-Free Survival in Prespecified Subgroups eFigure 3. Overall Survival in Prespecified Subgroups eFigure 4. Disease-Free Survival and Overall Survival by Treatment Group in Axillary Nodal Metastasis Categories [file jamanetwopen-e2429772-s002.pdf]

Supplementary Online Content

Joensuu H, Fraser J, Wildiers H, et al. Long-term outcomes of adjuvant trastuzumab for 9 weeks or 1 year for *ERBB2*-positive breast cancer: a secondary analysis of the SOLD randomized clinical trial. *JAMA Netw Open*. 2024;7(8):e2429772. doi:10.1001/jamanetworkopen.2024.29772

eTable 1. Multivariable Cox Proportional Hazards Regression Model for Disease-Free Survival
eTable 2. Multivariable Cox Proportional Hazards Regression Model for Overall Survival
eFigure 1. CONSORT Diagram of the Trial
eFigure 2. Disease-Free Survival in Prespecified Subgroups
eFigure 3. Overall Survival in Prespecified Subgroups
eFigure 4. Disease-Free Survival and Overall Survival by Treatment Group in Axillary Nodal Metastasis Categories

This supplementary material has been provided by the authors to give readers additional information about their work.

© 2024 Joensuu H et al. *JAMA Network Open*.

eTable 1. A Cox Multivariable Model for Disease-free Survival

**Covariable**	**HR (95% CI)**	***P***
Age at study entry (continuous)	1.01 (1.00-1.02)	.06
Docetaxel starting dose (80 mg/m^2 vs 100 mg/m^2)	1.05 (0.81-1.38)	.70
Estrogen receptor status (negative vs. positive)	1.10 (0.88-1.37)	.40
Number of positive axillary nodes (4+ vs 1-3 vs 0)	2.28 (1.65-3.15)	<.001
Stage (2 or 3 vs 1)	1.53 (1.13-2.08)	.006
Treatment group (9 weeks vs 1 year)	1.36 (1.10-1.68)	.005

Abbreviations: HR, hazard ratio; CI, confidence interval.

© 2024 Joensuu H et al. *JAMA Network Open*.

eTable 2. A Cox Multivariable Model for Overall Survival

**Covariable**	**HR (95% CI)**	***P***
Age at study entry (continuous)	1.03 (1.01-1.05)	<0.001
Docetaxel starting dose (80 mg/m^2 vs 100 mg/m^2)	0.98 (0.66-1.44)	0.90
Estrogen receptor status (negative vs. positive)	1.06 (0.78-1.45)	0.70
Number of positive axillary nodes (4+ vs 1-3 vs 0)	2.77 (1.79-4.30)	<.001
Stage (2 or 3 vs 1)	1.93 (1.22-3.03)	.005
Treatment group (9 weeks vs 1 year)	1.22 (0.90-1.64)	0.20

Abbreviations: HR, hazard ratio; CI, confidence interval.

© 2024 Joensuu H et al. *JAMA Network Open*.

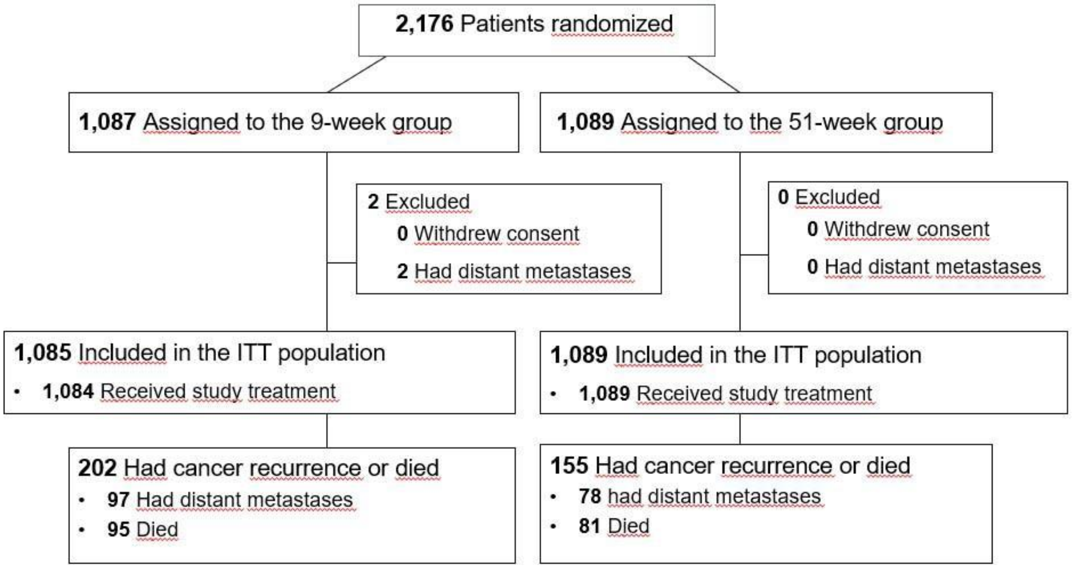

eFigure 1. The CONSORT diagram of the trial. ITT, intention-to-treat.

© 2024 Joensuu H et al. *JAMA Network Open*.

Subgroup		Hazard Ratio (90-% CI)	p(interaction)
ER status	Positive	1.267 (1.016 - 1.581)	
	Negative	1.555 (1.161 - 2.082)	0.358
Docetaxel dose	80 mg/m2	1.576 (1.290 - 1.924)	
	100 mg/m2	0.759 (0.510 - 1.129)	0.007
Axillary nodes with cancer	0	1.278 (0.994 - 1.642)	
	1-3	1.638 (1.176 - 2.282)	
	>3	1.267 (0.874 - 1.836)	0.572
Age (years)	<50	1.179 (0.849 - 1.639)	
	>=50	1.435 (1.165 - 1.767)	0.408
Stage	I	1.503 (1.078 - 2.094)	
	II / III	1.291 (1.050 - 1.588)	0.524

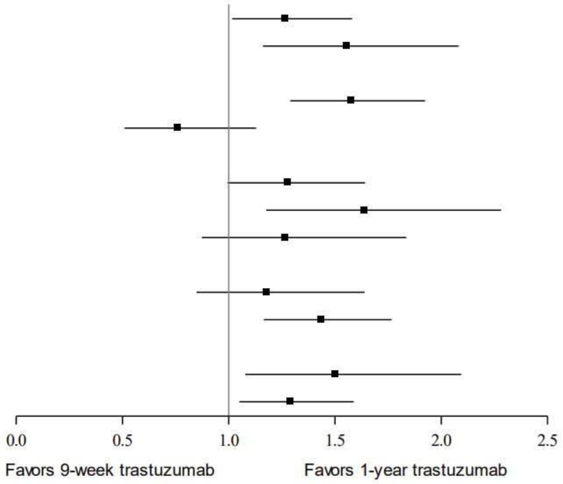

eFigure 2. Disease-free survival in prespecified subgroups. ER, estrogen receptor.

© 2024 Joensuu H et al. *JAMA Network Open*.

Subgroup		Hazard Ratio (90-% CI)	p(interaction)
ER status	Positive	1.129 (0.823 - 1.547)	
	Negative	1.380 (0.916 - 2.079)	0.522
Docetaxel dose	80 mg/m2	1.323 (1.000 - 1.752)	
	100 mg/m2	0.863 (0.485 - 1.533)	0.271
Axillary nodes with cancer	0	1.328 (0.907 - 1.943)	
	1-3	1.508 (0.944 - 2.408)	
	>3	0.788 (0.488 - 1.271)	0.231
Age (years)	<50	0.901 (0.550 - 1.476)	
	>=50	1.324 (0.990 - 1.770)	0.269
Stage	I	1.655 (0.963 - 2.844)	
	II / III	1.087 (0.821 - 1.441)	0.258

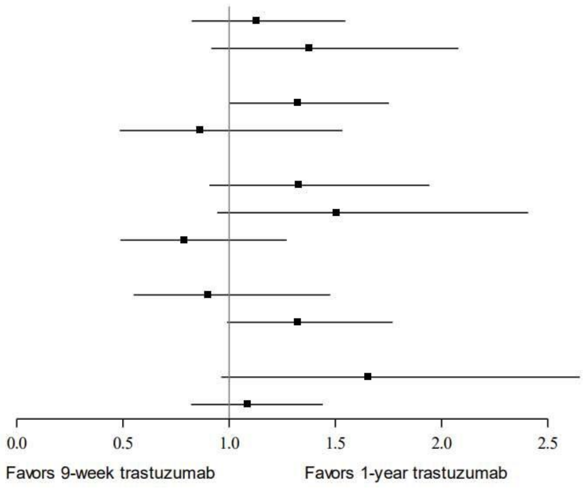

eFigure 3. Overall survival in prespecified subgroups. ER, estrogen receptor.

© 2024 Joensuu H et al. *JAMA Network Open*.

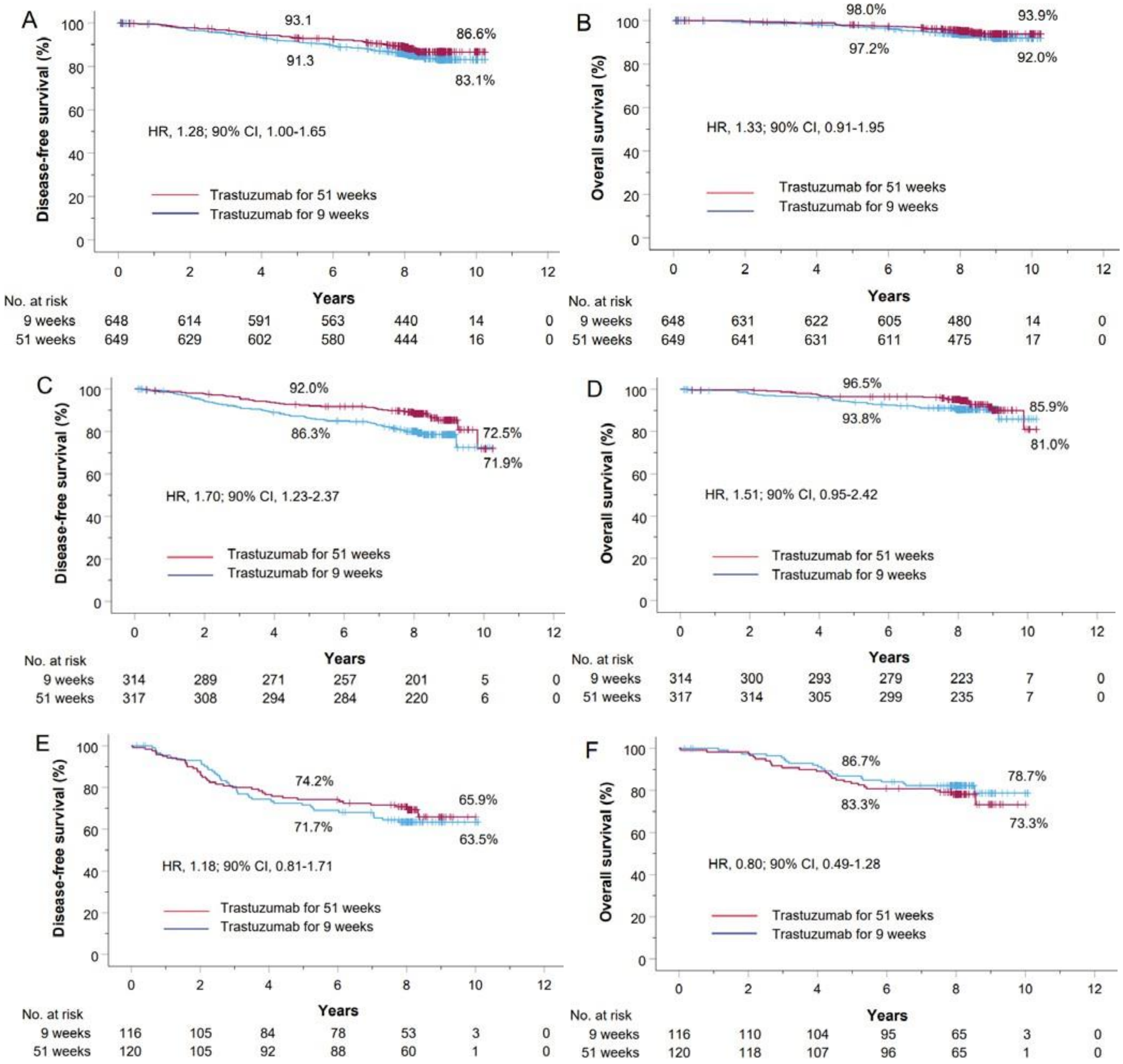

eFigure 4. Disease-free survival (panels A, C, and E) and overall survival (panels B, D, and F) by the treatment group in axillary nodal metastasis categories pN0 (A, B; no metastases), pN1-3 (C, D; 1-3 metastases), and pN4+ (E, F; 4 or more metastases). The patients without an event are indicated with a bar. HR, hazard ratio; CI, confidence interval.

© 2024 Joensuu H et al. *JAMA Network Open*.
