# Supplementary material for: Long-Term Outcomes of Adjuvant Trastuzumab for 9 Weeks or 1 Year for ERBB2-Positive Breast Cancer: A Secondary Analysis of the SOLD Randomized Clinical Trial
Source: JAMA Netw Open. 2024 Aug 26;7(8):e2429772. doi: 10.1001/jamanetworkopen.2024.29772 (PMC12507463; doi:10.1001/jamanetworkopen.2024.29772)
Supplement: Supplement 3. — Data Sharing Statement [file jamanetwopen-e2429772-s003.pdf]

Data Sharing Statement

Joensuu. Long-Term Outcomes of Adjuvant Trastuzumab for 9 Weeks or 1 Year for ERBB2-Positive Breast Cancer. *JAMA Netw Open*. Published August 26, 2024.
doi:10.1001/jamanetworkopen.2024.29772

Data
Data available: Yes
Data types: Deidentified participant data
How to access data: The trial data sets that support the current findings are not publicly available to protect patient privacy. The deidentified data will be available on a reasonable request from the corresponding author Heikki Joensuu; heikki.joensuu@hus.fi.
When available: With publication

Supporting Documents
Document types: None

Additional Information
Who can access the data: researchers whose proposed use of the data has been approved
Types of analyses: for a specified purpose: advancing treatment of cancer
Mechanisms of data availability: with investigator support
Any additional restrictions: the European Union General Data Protection Regulation
